# Tunneling current and noise of entangled electrons in correlated double quantum dot

**DOI:** 10.1038/s41598-021-88721-7

**Published:** 2021-04-29

**Authors:** N. S. Maslova, P. I. Arseyev, V. N. Mantsevich

**Affiliations:** 1 Quantum Technology Center and Quantum electronics department, Faculty of Physics, Lomonosov Moscow State University, Moscow, 119991 Russia; 2 P.N. Lebedev Physical Institute RAS, 119991 Moscow, Russia; 3 Quantum Technology Center and department of Semiconductor physics and Cryoelectronics, Faculty of Physics, Lomonosov Moscow State University, Moscow, 119991 Russia

**Keywords:** Condensed-matter physics, Semiconductors, Physics, Theoretical physics

## Abstract

We developed general approach for the analysis of tunneling current and its zero frequency noise for a wide class of systems where electron transport occurs through the intermediate structure with localized electrons. Proposed approach opens the possibility to study electron transport through multi-electron correlated states and allows to reveal the influence of spatial and spin symmetry of the total system on the electron transport. This approach is based on Keldysh diagram technique in pseudo-particle representation taking into account the operator constraint on the number of pseudo-particles, which gives the possibility to exclude non-physical states. It was shown that spatial and spin symmetry of the total system can block some channels for electron transport through the correlated quantum dots. Moreover, it was demonstrated that the stationary tunneling current and zero frequency noise in correlated coupled quantum dots depend on initial state of the system. In the frame of the proposed approach it was also shown that for the parallel coupling of two correlated quantum dots to the reservoirs tunneling current and its zero frequency noise are suppressed if tunneling occurs through the entangled triplet state with zero total spin projection on the z axis or enhanced for the tunneling through the singlet state in comparison with electron transport through the uncorrelated localized single-electron state. Obtained results demonstrate that two-electron entangled states in correlated quantum dots give the possibility to tune the zero frequency noise amplitude by blocking some channels for electron transport that is very promising in the sense of two-electron entangled states application in quantum communication and logic devices. The obtained nonmonotonic behavior of Fano factor as a function of applied bias is the direct manifestation of the possibility to control the noise to signal ration in correlated quantum dots. We also provide detailed calculations of current and noise for both single type of carriers and two different types of carriers in the presence and in the absence of Coulomb interaction in Supplementary materials.

## Introduction

Electronic current noise is of great importance in fundamental science, technology research and various applications. It can be used to probe fundamental quantum effects in electronic transport^1–5^, and can be also treated as undesirable effect in electronic devices as it prevents observing the measured signal. The role of fluctuations increases with a decrease in the system size and dimensionality, making current and noise in nanosystems very prominent^6–11^. In nanoscale junctions tunneling current noise can appear both due to the applied bias voltage or to the temperature gradient in the contact leads^2,12,13^. When a device is voltage biased, non-equilibrium fluctuations become dominant and the shot-noise appears in the system^14^. Moreover, inter-particle interactions can strongly modify the noise spectrum^15–17^. In the absence of Coulomb correlations tunneling current noise through the intermediate system with localized electrons was studied both experimentally and theoretically^18–21^. The zero frequency noise in general case contains classical contribution and quantum corrections. It can be described by the Landauer formalism^2,22^. In this formalism the zero frequency noise is expressed through transmission probabilities for each tunneling channel.

Finite frequency quantum noise determines the light emission spectra in atomic scale tunneling contacts^23–29^. In the absence of electron-phonon and Coulomb interaction the edge of noise and light (plasmonic) emission spectrum is given by the value of applied bias (noise spectrum amplitude vanishes when frequency exceeds the value of applied bias). In the presence of inter-particle interaction the spectrum broadens and its edge position depends on the type of interaction^23,30^.

Recent experiments and theoretical investigations indicated that the processes involving interaction of tunneling electrons with plasmon-polaritons excited in the leads result in photon emission with overbias energies. Besides the influence of Coulomb correlations and electron-phonon interaction on finite frequency noise spectrum they also affect the properties of zero frequency noise^31–35^. The simplest structures for zero frequency noise properties investigation are single or double correlated quantum dots localized in the tunneling contact. For two correlated quantum dots tunneling current and zero frequency noise depend on the contact geometry and results are quite different for sequential and parallel configurations^36^. A sequential transport in a system of two strongly coupled quantum dots was studied in^37^. It was shown that the shot noise in the system is very sensitive to the internal electronic level structure of the coupled dot system and its specific coupling to the electrodes. In the Coulomb blockade regime super-Poissonian noise appears. In this case the Fano factor takes values larger than unity^8,38^. The enhanced Fano factor can also be found in symmetric systems inside the Coulomb blockade region where the current is much suppressed^39,40^. In the Coulomb-coupled double quantum dots noise spectrum can be used as a possible indicator of the entanglement in the transport experiments^41^ as shot-noise spectrum exhibits resonances at the transition frequencies of the system and contains useful information on its relaxation and dephasing properties^42–44^. In^34^ authors demonstrated that in a weak dissipation regime the dephasing and relaxation rates of the two-level system can be extracted from noise measurements. Contrary, in the strong dissipation regime the localization-delocalization transitions becomes visible in the low-frequency noise. The zero frequency noise in the system of two weakly coupled quantum dots in both configurations was analyzed in the regime, when applied bias is much smaller than the tunneling widths of localized electrons energy levels^35^. As interaction between quantum dots is weak, they can not be considered as a single complex with its own set of multi-electron states. So, Kondo correlations between the localized and conduction electrons in the leads can influence on the zero frequency noise. In this case current noise was analyzed using slave-boson mean-field approach^45,46^. In such an approach even the constraint on the possible physical states is taken into account only for the averaged pseudo-particle occupation numbers.

For modern electronic devices the situation when quantum dots are strongly coupled and there exists a full set of multi-electron states is of great interest. In such system there are single-electron and multi-electron states with particular spin and spatial symmetry, which result in a well defined selection rules for electron’s transitions. The most interesting states in coupled quantum dots system are two-electron states with opposite spins, which can form singlet state (total spin is zero) and triplet state (total spin is unity)^47^. Such states are entangled spin states and they can be well initialized, investigated and processed experimentally^48,49^. Pairwise entanglement between electrons in coupled quantum dots can be detected in two mesoscopic wires by measurement of the current noise in one of the wires as it was shown in^50^. The possibility to suppress shot noise in tunneling junction was demonstrated in^1,24,51^. In^52^ authors performed configuration interaction calculations on a singlet-triplet double quantum dot and revealed the possibility to switch the system between different regimes by tuning the interdot bias. Switching between different transport regimes also results in the noise characteristics of the studied system. The measurements of the cross correlations between temporal current fluctuations in two capacitively coupled quantum dots in the Coulomb blockade regime were performed in^53^. It was shown that the sign of the cross-spectral density can be tuned by both gate voltage and source-drain bias.

In the present paper we developed a general approach for analysis of tunneling current and its noise spectra for a wide class of systems where electron transport occurs through the intermediate structure with localized electrons. Proposed approach opens the possibility to study electron transport through multi-electron correlated states and allows to consider the influence of spatial and spin symmetry of the total system on its tunneling characteristics. This method is based on Keldysh diagram technique in pseudo-particle representation taking into account operator constraint on the number of pseudo-particles, which gives the possibility to exclude non-physical states. In the frame of proposed approach we demonstrated that for the parallel coupling of two interacting quantum dots with Coulomb correlations to the reservoirs tunneling current and its zero frequency noise are suppressed if tunneling occurs through the triplet state with zero spin projection on the z axis or enhanced for the tunneling through the singlet state in comparison with electron transport through the uncorrelated localized electron state.

The suggested generalized approach allows both to reproduce well known exact results for tunneling through a single-level uncorrelated quantum dot and to analyze beyond the mean-field approximation more complicated systems with strong Coulomb correlations. It also gives the possibility to determine the contribution of each multi-electron channel to tunneling current and its noise spectrum.

## Theoretical model

**Figure 1:**
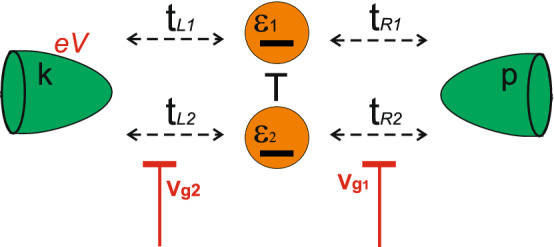
Scheme of two interacting quantum dots (impurity atoms) symmetrically coupled to reservoirs.

We consider a well-known system of two coupled single-level quantum dots (impurity atoms) connected symmetrically to two electronic reservoirs (see Fig. 1)^54^. The Hamiltonian of the system,

1 $$\begin{aligned} \hat{H}=\hat{H}_{dot}+\hat{H}_{res}+\hat{H}_{tun} \end{aligned}$$

is written as a sum of the Hamiltonian describing the quantum dots

2 $$\begin{aligned} \hat{H}_{dot}=\sum _{l\sigma }\varepsilon _l\hat{c}_{l\sigma }^{\dag }\hat{c}_{l\sigma }+\sum _{l\sigma }U_{l}\hat{n}_{l}^{\sigma }\hat{n}_{l}^{-\sigma } +T\sum _{\sigma }(\hat{c}_{1\sigma }^{\dag }\hat{c}_{2\sigma }+\hat{c}_{2\sigma }^{\dag }\hat{c}_{1\sigma }),\nonumber \\ \end{aligned}$$

the reservoir part

3 $$\begin{aligned} \hat{H}_{res}=\sum _{p\sigma }\varepsilon _p\hat{c}_{p\sigma }^{\dag }\hat{c}_{p\sigma }+\sum _{k\sigma }(\varepsilon _k-eV)\hat{c}_{k\sigma }^{\dag }\hat{c}_{k\sigma }, \end{aligned}$$

and the tunneling Hamiltonian

4 $$\begin{aligned} \hat{H}_{tun}=\sum _{lp\sigma }t_{Ll}(\hat{c}_{p\sigma }^{\dag }\hat{c}_{l\sigma }+\hat{c}_{l\sigma }^{\dag }\hat{c}_{p\sigma })+ \sum _{lk\sigma }t_{Rl}(\hat{c}_{k\sigma }^{\dag }\hat{c}_{l\sigma }+\hat{c}_{l\sigma }^{\dag }\hat{c}_{k\sigma }),\nonumber \\ \end{aligned}$$

where $\varepsilon _l$ ($l = 1,2$) is the spin-degenerate single-electron level and $U_l$ is the on-site Coulomb repulsion for double occupation of each quantum dot. The creation/annihilation of an electron with spin $\sigma =\pm 1$ within a dot is denoted by operators $\hat{c}_{l\sigma }^{\dag }/\hat{c}_{l\sigma }$ and $\hat{n}_{l}^{\sigma }=\hat{c}_{l\sigma }^{\dag }\hat{c}_{l\sigma }$ is the corresponding occupation number operator. Operator $\hat{c}_{k(p)\sigma }^{\dag }/\hat{c}_{k(p)\sigma }$ creates (annihilates) an electron with spin $\sigma$ and momentum *k*(*p*) in the reservoir. Coupling between the dots *T* and the tunneling transfer amplitudes to the reservoirs $t_{Ll}$ and $t_{Rl}$ are considered to be independent of momentum and spin. *eV* is an external bias voltage applied to one of the reservoirs. For simplicity we consider nearly identical quantum dots ($\varepsilon _1 = \varepsilon _2 = \varepsilon$ and $U_1 = U_2 = U$) and assume the symmetric coupling to both reservoirs: electrons can transfer from both quantum dots to the reservoirs and back with the same tunneling amplitude $t_{L(R)1}=t_{L(R)2}$.

When interaction between quantum dots exceeds coupling strength to the reservoir ($T \gg t_{L(R)1},t_{L(R)2}$), it is reasonable to use the basis of exact eigenfunctions and eigenvalues of coupled quantum dots neglecting interaction with the reservoirs. In this case all energies of single- and two-electron states are well known (further we will not consider three- and four-electron states):

In the case of one electron in the system, there exist two single-electron states with energies $\varepsilon _i=\varepsilon \pm T$ ($i=a,S$) and the wave function

5 $$\begin{aligned} \Psi _{i}^{\sigma }=\mu _{i}|0\uparrow \rangle |00\rangle +\nu _{i}|00\rangle |0\uparrow \rangle , \end{aligned}$$

where $|0\uparrow \rangle |00\rangle$ and $|00\rangle |0\uparrow \rangle$ are the basis functions corresponding to the presence of a single electron in each quantum dot. For resonant case in the symmetric (antisymmetric) state the coefficients in expression (5) read $\mu _{S(a)}=\pm \nu _{S(a)}=\frac{1}{\sqrt{2}}$. There also exist six two-electron states: two of them are states with the same spin projection $T^{+}=|\uparrow 0\rangle |\uparrow 0\rangle$; $T^{-}=|\downarrow 0\rangle |\downarrow 0\rangle$, which correspond to the existence of two electrons localized in different quantum dots and four states with the opposite spin projections: functions $|\uparrow \downarrow \rangle |00\rangle$; $|00\rangle |\uparrow \downarrow \rangle$ describe two electrons localized in the same dot with the opposite directions of spin, and functions $|\downarrow 0\rangle |0\uparrow \rangle$; $|0\uparrow \rangle |\downarrow 0\rangle$ correspond to electrons localized in different dots. The two-electron wave function reads:

6 $$\begin{aligned} \Psi _{j}^{\sigma -\sigma }=\alpha _j|\uparrow \downarrow \rangle |00\rangle +\beta _j|\downarrow 0\rangle |0\uparrow \rangle + \gamma _j|0\uparrow \rangle |\downarrow 0\rangle +\delta _j|00\rangle |\uparrow \downarrow \rangle . \end{aligned}$$

For low energy and excited singlet and triplet states coefficients $\alpha _j$, $\beta _j$, $\gamma _j$ and $\delta _j$ are determined by the eigenvalues and eigenvectors of 4*x*4 matrix of $\hat{H}_{dot}$ Hamiltonian in the basis $|\uparrow \downarrow \rangle |00\rangle$, $|\downarrow 0\rangle |0\uparrow \rangle$, $|0\uparrow \rangle |\downarrow 0\rangle$ and $|00\rangle |\uparrow \downarrow \rangle$:

7 $$\begin{aligned} \begin{pmatrix} 2\varepsilon +U &{}&{}\quad -T &{}&{}\quad -T &{}&{}\quad 0 \\ -T &{}&{}\quad 2\varepsilon &{}&{}\quad 0 &{}&{}\quad -T\\ -T &{}&{}\quad 0 &{}&{}\quad 2\varepsilon &{}&{}\quad 0\\ 0 &{}&{}\quad -T &{}&{}\quad -T &{}&{}\quad 2\varepsilon +U\end{pmatrix}. \end{aligned}$$

These are low energy singlet $S^{0}$ and triplet $T^{0}$ states and excited singlet ($S^{0*}$) and triplet ($T^{0*}$) states. For the triplet initial state $T^{0}$ coefficients $\alpha =\delta =0$ and $\beta =-\gamma =\frac{1}{\sqrt{2}}$. So, four two-electron states with opposite spins have the following energies: $2\varepsilon$; $2\varepsilon +U$ and $2\varepsilon +\frac{U}{2}\pm \sqrt{\frac{U^{2}}{4}+4T^{2}}$.

Further, we will consider only the lower energy singlet $S^{0}$ and triplet $T^{0}$ states because excited $S^{0*}$ and $T^{0*}$ states are separated by Coulomb gap. One can also exclude states $T^{\pm }$ at low temperature (in all the calculations temperature is considered to be $0.01\varepsilon$) by introducing weak exchange interaction with constant $J_{z}>0$ in the following form:

8 $$\begin{aligned} \hat{H}_{ex}=J_{z}(\hat{n}_{1}^{\sigma }-\hat{n}_{1}^{-\sigma })(\hat{n}_{2}^{\sigma }-\hat{n}_{2}^{-\sigma }). \end{aligned}$$

Correlated quantum dots kinetics can be analyzed by means of the pseudo-particle formalism^45,46^. In this theoretical approach, pseudo-particles are introduced for each eigenstate of the system. As we neglect excited double-occupied electron states as well as three- and four-particle states due to the presence of Coulomb correlations, the electron operator $\hat{c}_{l\sigma }^{\dag }$ $(l=1,2)$ should be rewritten as a combination of pseudo-particle operators:

9 $$\begin{aligned} \hat{c}_{l\sigma }^{\dag }= & {} \sum _{i}A_{i}^{\sigma l}\hat{f}_{i\sigma }^{\dag }\hat{b}+\sum _{ji\sigma }B_{ji}^{\sigma -\sigma l}\hat{d}_{j}^{\dag \sigma -\sigma }\hat{f}_{i-\sigma }+\sum _{i\sigma }B_{i}^{\sigma \sigma l}\hat{d}^{\dag \sigma \sigma }\hat{f}_{i\sigma }, \end{aligned}$$

where $\hat{f}_{i\sigma }^{+}(\hat{f}_{i\sigma })$ are pseudo-fermion creation (annihilation) operators for electronic states with one electron and $\hat{b}^{\dag }(\hat{b})$, $\hat{d}^{\dag \sigma \sigma }(\hat{d}^{\sigma \sigma })$, $\hat{d}_{j}^{\dag \sigma -\sigma }(\hat{d}_{j}^{\sigma -\sigma })$ are slave-boson operators, corresponding to the states without electrons or with two electron. $A_{i}^{\sigma l}$, $B_{ji}^{\sigma -\sigma l}$ and $B_{i}^{\sigma \sigma l}$ are matrix elements of the creation operators $\hat{c}_{l\sigma }^{\dag }$ between the states with *n* and $n+1$ electrons^17^. Constraint on the possible physical states has the form

10 $$\begin{aligned} \hat{N}_{b}+\sum _{i\sigma }\hat{N}_{i\sigma }+\sum _{j\sigma \sigma ^{'}}\hat{N}_{j}^{\sigma \sigma ^{'}}=1. , \end{aligned}$$

where $\hat{N}_{b}=\hat{b}^{\dag }\hat{b}$, $\hat{N}_{i\sigma }=\hat{f}_{i\sigma }^{\dag }\hat{f}_{i\sigma }$ and $\hat{N}_{j}^{\sigma -\sigma }=\hat{d}_{j}^{\dag \sigma -\sigma }\hat{d}_{j}^{\sigma -\sigma }$. As we excluded $T^{\pm }$ states, terms containing operator $\hat{d}^{\dag \sigma \sigma }$ in expressions (9)–(10) should be also omitted. In pseudo-particle representation intermediate system Hamiltonian $\hat{H}_{dot}$ has the form of non-interacting pseudo-particles:

11 $$\begin{aligned} \hat{H}_{dot}=\sum _{i\sigma }\varepsilon _i\hat{f}_{i\sigma }^{\dag }\hat{f}_{i\sigma }+\sum _{j=S^0,T^0}E_{j}^{\sigma -\sigma }\hat{d}_{j}^{\dag \sigma -\sigma }\hat{d}_{j}^{\sigma -\sigma } \end{aligned}$$

with $E_{j}^{\sigma -\sigma }$ being the two-electron states energies. All the correlations are now included in the constraint (10). Interaction with the leads becomes nonlinear in pseudo-particle approach and can be obtained by substituting expression for electron operators given by Eq. (9) in the tunneling Hamiltonian (4). We set $\hbar =1$ and $e=1$ in what follows. The tunneling current operator is

12 $$\begin{aligned} \hat{I}_{L}(t)=\sum _{k}\dot{\hat{n}}_{k} =t_{L}\sum _{k,l,\sigma }[\hat{c}_{k\sigma }^{\dag }(t)\hat{c}_{l\sigma }(t)-h.c.]. \end{aligned}$$

The current noise is characterized by the set of correlation functions

13 $$\begin{aligned} S_{\alpha \beta }(t,t')= & {} <\hat{I}_{\alpha }(t){\hat{I}_{\beta }(t')}>-<\hat{I}_{\alpha }(t)> <{\hat{I}_{\beta }(t')}>, \end{aligned}$$

where indexes $\alpha ,\beta =L,R$. In expressions for tunneling current operator and current noise in pseudo-particle representation all localized electron operators $\hat{c}_{l\sigma }$ should be replaced using expression (9) with constraint (10)

14 $$\begin{aligned} \hat{I}_{L}(t)=\sum _{k,i,\sigma }t_{Li}\hat{f}^{\dag }_{i\sigma }(t)\hat{b}(t)\hat{c}_{k\sigma }+\sum _{k,i,j,\sigma ,\sigma '}t_{Lij}\hat{d}_{j}^{\dag \sigma \sigma '}\hat{f}_{i\sigma '}\hat{c}_{k\sigma }-h.c.,\nonumber \\ \end{aligned}$$

where

15 $$\begin{aligned} t_{Li}= & {} t_{L}\left( \mu _{i}+\nu _{i}\right) ,\nonumber \\ t_{Lij}= & {} t_{L}\left( \alpha _j\mu _{i}+\beta _j\nu _{i}+\delta _j\nu _{i}+\gamma _j\mu _{i}\right) \end{aligned}$$

with indexes $i=S,a$ and $j=S^0,T^0$. For the resonant case, when $\varepsilon _1=\varepsilon _2$ the following expressions for tunneling amplitudes are valid for a single electron states $t_{La}=0$ and $t_{LS}=\sqrt{2}t_L$. Tunneling amplitude from symmetric single-electron state is enhanced due to constructive interference between electrons. For two-electron states one can obtain $t_{LT^0a}=t_L$, $t_{LT^0S}=t_{LS^0a}=0$ and $t_{LS^0S}=\sqrt{2}t_L (\alpha +\beta )$. So, two independent tunneling channels appear: the first one through the triplet state $T^0$ and antisymmetric single electron state *a* and the second one through the singlet state $S^0$, symmetric single electron state *S* and empty state. In the case of tunneling through the $T^0$ state one of the electrons with spin $\sigma$ can transfer to the lead and coupled quantum dots system remains in the antisymmetric single electron state *a* with the opposite spin $-\sigma$. This single electron in antisymmetric state *a* can not transfer to the lead because transitions between antisymmetric and empty states are restricted by the selection rules. For the singlet $S^0$ state electron with spin $\sigma$ can transfer to the lead leaving the second electron with opposite spin in the symmetric single electron state *S*. Further, this electron can also tunnel to the lead and quantum dots system remains in the empty state.

It follows from the general principles of statistical physics that the correlation function in the steady state depends only on the difference of times $\tau =t'-t$. It is instructive to introduce the Fourier transform of the correlation function in the form

16 $$\begin{aligned} S_{\alpha \beta }(\omega )=\int _{-\infty }^{\infty }S_{\alpha \beta }(t,t+\tau )e^{i\omega \tau }d\tau , \end{aligned}$$

where $\alpha ,\beta =L,R$. Although in the steady state the current through the structure is the same, the current fluctuations are not homogeneous in space in general. Hence, generally in the presence of intermediate system $S_{LL}\ne S_{RR}\ne S_{LR}\ne S_{RL}$, although at $\omega =0$ all components are the same^17^.

## Current and noise in the system of two coupled QDs interacting with the reservoir

We now analyze current and zero frequency noise for two quantum dots symmetrically coupled to both leads $t_{L(R)1}=t_{L(R)2}=t_{L(R)}$ (see Fig. 1). Further for simplicity we will consider resonant case $\varepsilon _1=\varepsilon _2=\varepsilon$. Symmetric properties of the total system results in the selection rules for electron transitions determined by the corresponding matrix elements. This results in the following expressions for the tunneling rates. The tunneling rate between single-occupied antisymmetric state *a* and empty state in the case of symmetric coupling is $\gamma _{L(R)}^{a}=\gamma _{L(R)}|\mu _a+\nu _a|^{2}=0$, $\gamma _{L(R)}=\pi t_{L(R)}^{2}\nu _{L(R)}$. The tunneling rate between double-occupied singlet state $S^0$ and antisymmetric state *a* is $\gamma _{L(R)}^{S^{0}a}=|\alpha +\beta |^{2}\gamma _{L(R)}^{a}=0$. So, in this case only transitions between $T^0$ and $a^{\pm }$ states are allowed. For electron transitions between $T^0$ and $a^{\pm }$ states tunneling rates are $\gamma _{L(R)}^{T^{0}a}=\gamma _{L(R)}|\beta _{T^0}\nu _a+\gamma _{T^0}\mu _a|^{2}=\gamma _{L(R)}$. For single-electron symmetric two-electron states *S* tunneling rates are defined as: $\gamma _{L(R)}^{S^0S}=\gamma _{L(R)}|\alpha +\beta |^{2}2$, $\gamma _{L(R)}^{S}=\gamma _{L(R)}|\mu _S+\nu _S|^{2}=2\gamma _{L(R)}$ and $\gamma _{L(R)}^{S^0S}=0$. So, there exist two independent channels for tunneling: $T^0-a$ channel corresponds to the electron transitions in coupled quantum dots between two-electron triplet state $T^0$ and antisymmetric single-electron state *a*, and $S^0-S-0$ channel corresponds to the electron transitions between two-electron singlet state $S^0$, symmetric single electron state *S* and empty state. We will consider the situation when applied bias voltage *eV* strongly exceeds all the tunneling rates, so the Kondo effect is not significant for such a situation. First of all we consider the system to be initially prepared in a single electron antisymmetric state. It can be done by means of the external laser pulse, when the resonant excitation of the coupled quantum dots system occurs (the frequency of the laser pulse is in the resonance with the antisymmetric single electron state energy level, consequently, the $\varepsilon _a$ state becomes occupied)^54^. Another possibility to prepare system in the $\varepsilon _a$ state is to use gates structure. Changing the gate voltage one can organize the situation when at the initial time moment antisymmetric single electron state is localized below the Fermi level and, consequently, is occupied. Further tuning of gate voltage results in the Fermi level position changing in such a way that it is localized below the $\varepsilon _a$ energy level. In this case tunneling current flows through the $T^0-a$ channel. For calculation of tunneling current and zero frequency noise in $T^0-a$ channel we determine pseudo-particle Green’s functions $T^{0<}=-N^{T^{0}}(T^{0A}-T^{0R})$, $N^{<}_{a\pm \sigma }=N^{a}(N^{0A}_{a\pm \sigma }-N^{0R}_{a\pm \sigma })$ and $T^{0R(A)}=\frac{1}{\omega -E_{T^0}\pm i\gamma ^{T^0a}}$, $N^{0R(A)}_{a\pm \sigma }=\frac{1}{\omega -\varepsilon _a\pm i\gamma ^{T^0a}}$. The pseudo-particle occupation numbers can be found from kinetic equations shown in Supplementary Appendix I:

17 $$\begin{aligned} N^{T^{0}}= & {} \frac{N_T^{T^{0}a}}{2-N_T^{T^{0}a}},\nonumber \\ N^{a}= & {} \frac{1-N_T^{T^{0}a}}{2-N_T^{T^{0}a}}, \end{aligned}$$

where $N_T^{T^{0}a}=\frac{\gamma _{L}^{T^{0}a}\Phi _{k}^{T^{0}a}+\gamma _{R}^{T^{0}a}\Phi _{p}^{T^{0}a}}{\gamma _{L}^{T^{0}a}+\gamma _{R}^{T^{0}a}}$ and $N_T^{T^{0}a}\equiv N_T(E_{T^{0}}-\varepsilon _a)$. Functions $\Phi _{k(p)}^{T^{0}a}\equiv \Phi _{k(p)}(E_{T^{0}}-\varepsilon _a)$ read

18 $$\begin{aligned} \Phi _{k(p)}(E_{T^{0}}-\varepsilon _a)= {} \frac{1}{2\pi }i\int d\varepsilon _{k(p)}f_{k(p)}(\varepsilon _{k(p)})\times \left[ \frac{1}{E_{T^{0}}-\varepsilon _a+i(\gamma _{L}+\gamma _R)-\varepsilon _{k(p)}}\right. \left. -\frac{1}{E_{T^{0}}-\varepsilon _a-i(\gamma _{L}+\gamma _R)-\varepsilon _{k(p)}}\right] \end{aligned}$$

with energy of the triplet state being $E_{T^{0}}=2\varepsilon$ and $f_{k(p)}$—is the Fermi distribution function of electrons in the lead *k*(*p*).

**Figure 2:**
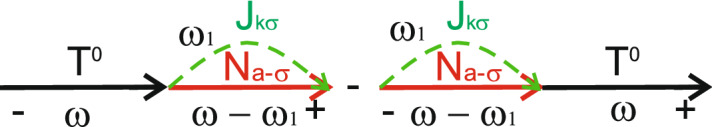
Diagrams contributing to the tunneling current through the $T^0-a$ channel.

Tunneling current is determined by the diagrams shown in Fig. 2. The real electron Green’s functions in the leads for a given spin $\sigma$ are determined as:

19 $$\begin{aligned} J_{k\sigma }^{<(>)}(\omega )= & {} \int d\epsilon _k G_{k\sigma }^{<(>)}(\omega ,k),\nonumber \\ G_{k\sigma }^{<}(\omega )= & {} f_{k\sigma }(\omega )\left[ G_{k\sigma }^{A}(\omega )-G_{k\sigma }^{R}(\omega )\right] ,\nonumber \\ G_{k\sigma }^{>}(\omega )= & {} (f_{k\sigma }(\omega )-1)\left[ G_{k\sigma }^{A}(\omega )-G_{k\sigma }^{R}(\omega )\right] ,\nonumber \\ J_{T\sigma }^{<(>)}(\omega )= & {} \frac{\gamma _LJ_{k\sigma }^{<(>)}(\omega )+\gamma _RJ_{p\sigma }^{<(>)}(\omega )}{\gamma _L+\gamma _R}. \end{aligned}$$

Details of tunneling current calculations for each spin channel for $eV \gg \gamma _{L(R)}$ are shown in Supplementary Appendix II. Expression for the tunneling current reads

20 $$\begin{aligned} I_{\sigma }^{T^0a}=\frac{4\gamma _{L}^{T^0a}\gamma _{R}^{T^0a}\left( \Phi _{k}^{T^0a}-\Phi _{p}^{T^0a}\right) }{\gamma ^{T^0a}\left( 2-N_{T}^{T^0a}\right) }, \end{aligned}$$

where $$\gamma ^{T^0a}=\gamma _{L}^{T^0a}+\gamma _{R}^{T^0a}$$.

**Figure 3:**
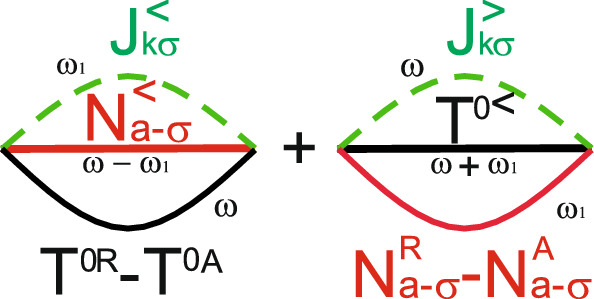
Leading diagrams contributing to the zero frequency noise in the $T^0-a$ channel.

**Figure 4:**
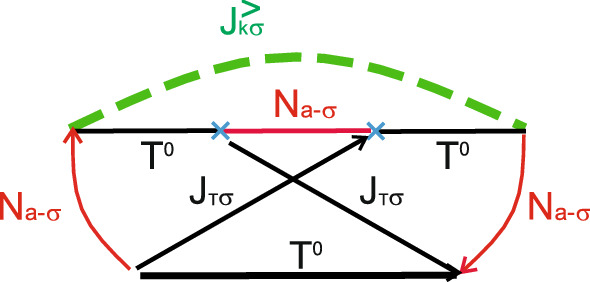
Maximally crossing diagrams contributing to the zero frequency noise quantum corrections in the $T^0-a$ channel.

Similarly one can obtain expression for the zero frequency noise spectra. The leading terms are given by the diagrams shown in Fig. 3. Quantum corrections contain maximally crossing diagrams, which are depicted in Fig. 4. Fixing the sign of spin index $\sigma$ for the Green’s function of electrons in the leads $J^{<}_{k\sigma }(\omega )$ automatically determines the signs of spin index $\sigma$ for all pseudo-particle Green’s functions $N_{\pm \sigma }$ and electron Green’s functions in the leads $J_{T\sigma }^{<(>)}$ in maximally crossing diagrams. Expression for the total zero frequency noise reads

21 $$\begin{aligned} S^{T^0a}(0)=\sum _{\sigma }S_{T^0a}^{\sigma }(0)=2 \frac{4\gamma _{L}^{T^0a}\gamma _{R}^{T^0a}}{\gamma ^{T^0a}(2-N_{T}^{T^0a})}\left[ 1-\frac{2\gamma _L^{T^0a}\gamma _R^{T^0a}}{(\gamma ^{T^0a})^2}\right] \left[ (\Phi _{k}^{T^{0}a})^2(1-\Phi _{p}^{T^{0}a})^2+(k\leftrightarrow p)\right] . \end{aligned}$$

When the energy difference between $T^0$ state and antisymmetric single electron state *a* is out of the interval [0, *eV*] ($E_{T^0}-\varepsilon _a<0$ and $E_{T^0}-\varepsilon _a>eV$) tunneling current does not flow through the $T^0-a$ channel ($I_{\sigma }^{T^0a}=0$ and $S^{T^0a}(0)=0$). If the energy difference between $T^0$ state and antisymmetric single electron state *a* belongs to the interval [0, *eV*] ($0<E_{T^0}-\varepsilon _a<eV$ and $(|E_{T^0}-\varepsilon _0|)/\gamma _{L(R)} \gg 1$) tunneling current is expressed as $I_{\sigma }^{T^{0}a}\sim \frac{I_{1}}{2-N_{T}^{T^0a}}$. Tunneling current $I_{1}$ is a current through the single-level quantum dot without Coulomb correlations for particular spin channel. The explicit expression for the tunneling current $I_{1}$ can be found in Supplementary Appendix II. In this limit one can also simplify expression for the tunneling current noise

22 $$\begin{aligned} S^{T^0a}(0)=\frac{2S_1(0)}{2-N_{T}^{T^0a}}. \end{aligned}$$

The zero frequency noise $S_1(0)$ corresponds to tunneling through the single-level quantum dot without Coulomb correlations for particular spin. The explicit expression can be found in Supplementary Appendix II. In the symmetry case between tunneling rates occupation number is $N_{T}^{T^{0}a}\sim 1/2$. So, for symmetric coupling tunneling current $I_{\sigma }^{T^0a}/\gamma ^{T^0a}\sim 2/3\cdot I_{1}/\gamma$ and $S^{T^0a}_{\sigma }(0)/\gamma ^{T^0a}\sim 2/3\cdot S_{1}(0)/\gamma$. When tunneling occurs through the channel formed by the triplet state $T^0$ and antisymmetric singly-occupied state *a* zero frequency tunneling current noise is suppressed in comparison with tunneling current noise obtained for tunneling through the single-level localized state.

Now let us consider tunneling current and zero frequency noise, when electron transitions occur between singlet two-electron state $S^0$, single-electron symmetric states $S^{\pm }$ and empty state ($S^0-S$ and $S-0$). Such transitions determine tunneling current and zero frequency noise if quantum dots are initially prepared in symmetric single-electron state *S*. Such initial state can be prepared by means of the laser pulse excitation and further switching on gate voltage, which governs coupling between the dots and the leads^54^. Due to symmetric properties of the total system in the resonant case ($\varepsilon _1=\varepsilon _2=\varepsilon$) only transitions between $S^0$, $S^{\pm }$ and empty state are allowed with corresponding tunneling rates $\gamma _{L(R)}^{S^0S}=2\gamma _{L(R)}|\alpha +\beta |^{2}$ and $\gamma _{L(R)}^{S}=\gamma _{L(R)}|\mu _S+\nu _S|^{2}=2\gamma _{L(R)}$. Tunneling rate for transitions between $S^{\pm }$ single-occupied state and empty state increases twice due to constructive interference of tunneling electrons from two quantum dots. Transitions between $S^0-a_{\pm }$ are forbidden due to the selection rules. For calculation of tunneling current and zero frequency noise in $S^0-S-0$ channel we determine pseudo-particle Green’s functions $S^{0<}=-N^{S^{0}}(S^{0A}-S^{0R})$, $N^{<}_{S\pm \sigma }=N^{S}(N^{0A}_{S\pm \sigma }-N^{0R}_{S\pm \sigma })$ and $S^{0R(A)}=\frac{1}{\omega -E_{S^0}\pm i\gamma ^{S^0S}}$, $N^{0R(A)}_{S\pm \sigma }=\frac{1}{\omega -\varepsilon _a\pm i\gamma ^{S^0S}}$. One should also introduce functions $B^{R(A)}=\frac{1}{\omega \pm i\gamma }$, $B^{<}=b(B^{R}-B^{A})$. The pseudo-particle occupation numbers can be found from kinetic equations shown in Supplementary Appendix I.

23 $$\begin{aligned} N^{S^0}= & {} \frac{N_T^{S}N_T^{S^0S}}{1+N_T^{S}-N_T^{S^0S}},\nonumber \\ N^{S}= & {} \frac{N_T^{S}(1-N_T^{S^0S})}{1+N_T^{S}-N_T^{S^0S}},\nonumber \\ b= & {} \frac{(1-N_T^{S})(1-N_T^{S^0S})}{1+N_T^{S}-N_T^{S^0S}}, \end{aligned}$$

where $N_T^{S^{0}S}=\frac{\gamma _{L}^{S^{0}S}\Phi _{k}^{S^{0}S}+\gamma _{R}^{S^{0}S}\Phi _{p}^{S^{0}S}}{\gamma _{L}^{S^{0}S}+\gamma _{R}^{S^{0}S}}$, $N_T^{S}=\frac{\gamma _{L}^{S}\Phi _{k}^{S}+\gamma _{R}^{S}\Phi _{p}^{S}}{\gamma _{L}^{S}+\gamma _{R}^{S}}$ and $N_T^{S^{0}S}\equiv N_T(E_{S^{0}}-\varepsilon _S)$, $N_T^{S}\equiv N_T(\varepsilon _S)$. Functions $\Phi _{k(p)}^{S^{0}S}\equiv \Phi _{k(p)}(E_{S^{0}}-\varepsilon _S)$ and $\Phi _{k(p)}^{S}\equiv \Phi _{k(p)}(\varepsilon _S)$ are determined by Eq. (18) with the corresponding energies changing.

**Figure 5:**
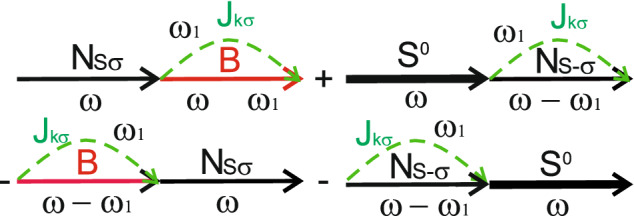
Diagrams contributing to the tunneling current through the $S^0-S-0$ channel.

Tunneling current in $S^0-S-0$ channel can be calculated using diagrams shown in Fig. 5. For each spin projection tunneling current reads

24 $$\begin{aligned} I_{\sigma }^{S^0-S-0}= I_{\sigma }^{S^0-S}+I_{\sigma }^{S-0}=&{} \frac{4\gamma _L^{S^0S}\gamma _R^{S^0S}}{\gamma ^{S^0S}(1+N_{T}^{S}-N_{T}^{S^0S})} N_{T}^{S}\left( \Phi _{k}^{S^0S}-\Phi _{p}^{S^0S}\right) \nonumber \\&+ \frac{4\gamma _L^{S}\gamma _R^{S}}{\gamma ^{S}(1+N_{T}^{S}-N_{T}^{S^0S})} \left( 1-N_{T}^{S^0S}\right) \left( \Phi _{k}^{S}-\Phi _{p}^{S}\right) ,\nonumber \\ \end{aligned}$$

where $\gamma ^{S}=\gamma _{L}^{S}+\gamma _{R}^{S}$ and $\gamma ^{S^0S}=\gamma _{L}^{S^0S}+\gamma _{R}^{S^0S}$. Calculation details are shown in Supplementary Appendix III.

**Figure 6:**
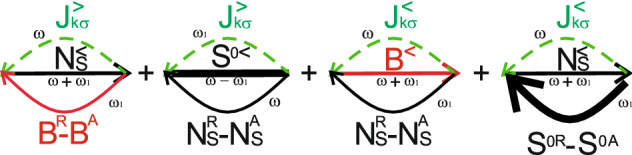
Leading diagrams contributing to the zero frequency noise in the $S^0-S-0$ channel.

**Figure 7:**
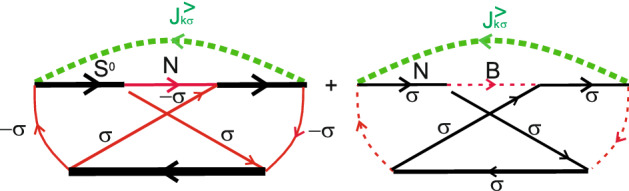
Maximally crossing diagrams contributing to the zero frequency noise quantum corrections in the $S^0-S-0$ channel.

Similarly, the zero frequency noise also has two contributions: intermediate system state changes between $S^0-S$ or between $S-0$, when electron tunnels to the leads. The leading terms in zero frequency noise for both contributions are shown in diagrams depicted in Fig. 6. Quantum corrections contain maximally crossing diagrams shown in Fig. 7. Fixing the sign of spin index $\sigma$ for the Green’s function of electrons in the leads $J^{<}_{k\sigma }(\omega )$ automatically determines the signs of spin index $\sigma$ for all pseudo-particle Green’s functions $N_{\pm \sigma }$ and electron Green’s functions in the leads $J_{\sigma }^{T<(>)}$ in maximally crossing diagrams. The detailed calculations are presented in Supplementary Appendix III. So, the expression for the zero frequency noise reads.

25 $$\begin{aligned} S^{S^0-S-0}_{\sigma }(0)=I_{\sigma }^{S^0-S}\left( 1-\frac{2\gamma _L^{S^0S}\gamma _R^{S^0S}}{(\gamma ^{S^0S})^{2}}\right) +I_{\sigma }^{S-0}\left( 1-\frac{2\gamma _L^{S}\gamma _R^{S}}{(\gamma ^{S})^{2}}\right) .\nonumber \\ \end{aligned}$$

For $E_{S^0}-\varepsilon _S>eV$ and $0<\varepsilon _S<eV$ only electron transitions between *S* and empty states contribute to tunneling current and noise. Thus:

26 $$\begin{aligned} I_{\sigma }^{S^0-S-0}= & {} 2I_{1}\frac{1-N_{T}^{S^0S}}{1+N_T^{S}-N_T^{S^0S}}\nonumber \\ \end{aligned}$$

and

27 $$\begin{aligned} S^{S_0-S-0}_{\sigma }(0)=2S_1(0)\frac{1-N_{T}^{S^0S}}{1+N_T^{S}-N_T^{S^0S}}. \end{aligned}$$

For symmetric tunneling contact ($\gamma _L=\gamma _R$) tunneling occupation numbers corresponding to the energy $E_{S^0}-\varepsilon _S$ aspire to zero ($N_T^{S^0S}\rightarrow 0$) and tunneling occupation number corresponding to the energy $\varepsilon _S$ is close to 1/2 ($N_T^{S}\rightarrow 1/2$). So, tunneling current and zero frequency noise can be estimated as $I_{\sigma }^{S^0-S-0}\sim 4/3\cdot I_{1}$ and $S^{\sigma }(0)\sim 4/3\cdot S_{1}(0)$.

When both the energy differences between the double-occupied singlet state $E_{S^0}$ and single-electron energy levels $\varepsilon _S$, $\varepsilon _a$ are in the interval [0, *eV*] ($0<E_{S^0}-\varepsilon _s, \varepsilon _s<eV$), both contributions to the tunneling current and zero frequency noise are significant: $I_{\sigma }^{S^0-S-0}=I_{\sigma }^{S-0}+I_{\sigma }^{S^0-S}$. In the limit of strong Coulomb interaction ($U \gg T$) coefficients $\alpha$ and $\beta$ can be estimated as $\alpha \ll 1$ and $\beta \sim \frac{1}{\sqrt{2}}$, so tunneling rates are of order of $\gamma _{L(R)}^{S^0S}\sim \gamma _{L(R)}^{S}/2$ and in this case

28 $$\begin{aligned} I_{\sigma }^{S-0}= & {} 2I_{1}\frac{1-N_{T}^{S^0S}}{1+N_T^{S}-N_T^{S^0S}},\nonumber \\ I_{\sigma }^{S^0-S}= & {} I_{1}\frac{N_{T}^{S}}{1+N_T^{S}-N_T^{S^0-S}}. \end{aligned}$$

Total tunneling current $I_{\sigma }^{S^0-S-0}$ is

29 $$\begin{aligned} I_{\sigma }^{S^0-S-0}=I_{1}+\frac{1-N_{T}^{S^0S}}{1+N_T^{S}-N_T^{S^0S}}I_{1}. \end{aligned}$$

Corresponding zero frequency noise $S_{\sigma }^{S^0-S-0}(0)$ reads

30 $$\begin{aligned} S_{\sigma }^{S^0-S-0}(0)=S_{1}(0)+\frac{1-N_{T}^{S^0S}}{1+N_T^{S}-N_T^{S^0S}} S_{1}(0).\nonumber \\ \end{aligned}$$

For symmetrical coupling ($\gamma _L=\gamma _R$) tunneling current and zero frequency noise can be estimated as: $I_{\sigma }^{S^0-S-0}\sim 3/2\cdot I_1$ and $S_{\sigma }^{S^0-S-0}(0)\sim 3/2\cdot S_1(0)$.

**Figure 8:**
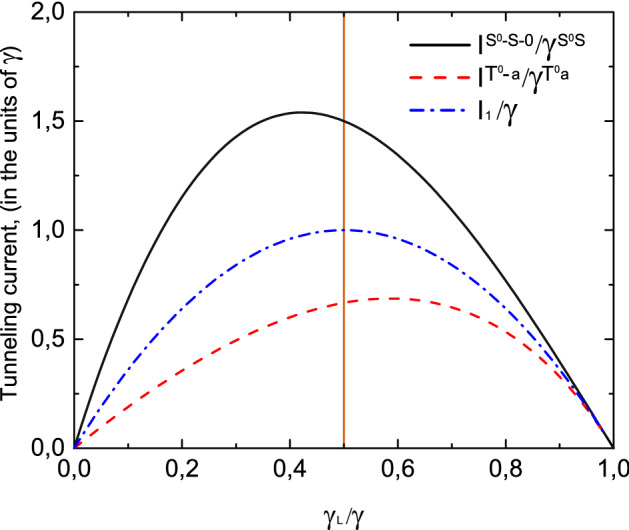
Tunneling current as a function of tunneling contact asymmetry ($\gamma _L/\gamma =\gamma _L^{T^0a}/\gamma ^{T^0a}=\gamma _L^{S^0S}/\gamma ^{S^0S}=\gamma _L^{S}/\gamma ^{S}$) for a single type of carriers $I_1/\gamma$ (blue dashed-dotted curve), for the $T^0-a$ channel $I^{T^0a}/\gamma ^{T^{0}a}$ (red dashed curve) and for the $S^0-S-0$ channel $I^{S^0-S-0}/\gamma ^{S^{0}S}$ (black solid curve). $U/T \gg 1$. Temperature is equal to $0.01\varepsilon$.

**Figure 9:**
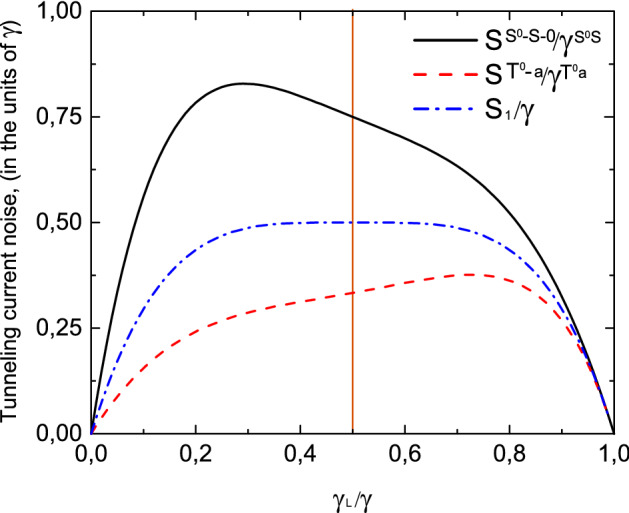
Zero frequency noise as a function of tunneling contact asymmetry ($\gamma _L/\gamma =\gamma _L^{T^0a}/\gamma ^{T^0a}=\gamma _L^{S^0S}/\gamma ^{S^0S}=\gamma _L^{S}/\gamma ^{S}$) for a single type of carriers $S_1/\gamma$ (blue dashed-dotted curve), for the $T^0-a$ channel $S^{T^0a}/\gamma ^{T^{0}a}$ (red dashed curve) and for the $S^0-S-0$ channel $S^{S^0-S-0}/\gamma ^{S^{0}S}$ (black solid curve). $U/T \gg 1$. Temperature is equal to $0.01\varepsilon$.

**Figure 10:**
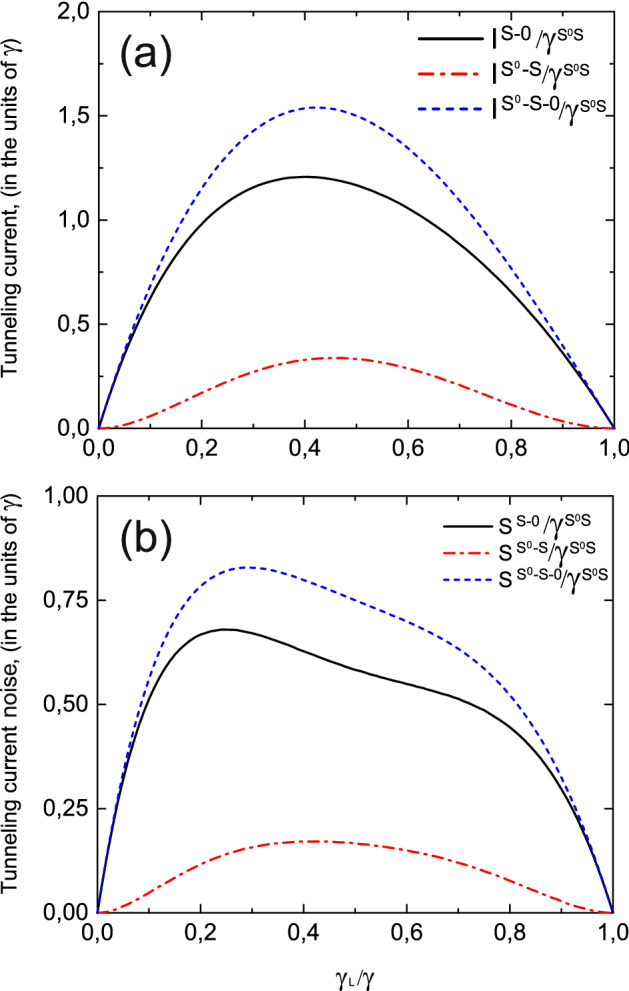
(**a**) Relative contributions $I^{S-0}/\gamma ^{S^{0}S}$ and $I^{S^0-S}/\gamma ^{S^{0}S}$ to the total tunneling current and total tunneling current $I^{S^0-S-0}/\gamma ^{S^{0}S}$; (**b**) Relative contributions $S^{S-0}/\gamma ^{S^{0}S}$and $S^{S^0-S}/\gamma ^{S^{0}S}$ to the total noise and total noise $S^{S^0-S-0}/\gamma ^{S^{0}S}$ as a functions of tunneling contact asymmetry ($\gamma _L/\gamma =\gamma _L^{S^0S}/\gamma ^{S^0S}$ and $\gamma ^{S^0S}=\gamma ^{S}/2$). $U/T \gg 1$. Temperature is equal to $0.01\varepsilon$.

Tunneling current through the coupled quantum dots and current noise spectra for $U/T \gg 1$ are depicted in Figs. 8 and 9 depending on the symmetry between the left and right leads—the ratio $\gamma _L/\gamma$. Tunneling rate $\gamma _L$ describes tunneling transfer between the filled states in the left lead and double quantum dot system. We performed a comparison with a simple case when tunneling current flows through the single level in the absence of Coulomb correlations. When the system is initially prepared in a single-electron antisymmetric state the electron transitions between triplet two-electron state $T^0$ and antisymmetric state *a* are allowed due to the selection rules based on the symmetry properties of the total system. So, only $T^0-a$ channel contributes to the tunneling current. As can be seen from Fig. 8 tunneling current is suppressed in comparison with the case when tunneling occurs through the single-level due to the destructive interference (see red dashed and blue dashed-dotted curves in Fig. 8). Moreover, the maximum value of tunneling current is achieved for asymmetric coupling with the leads when $\gamma _L/\gamma =2-\sqrt{2}$, contrary to the tunneling through the single-level, when maximum value corresponds to symmetric coupling with $\gamma _L/\gamma =1/2$. Zero frequency noise in the $T^0-a$ channel is also suppressed in comparison with the single-level case. The shift of its maximum value for the asymmetric coupling is more pronounced in comparison with the shift of the tunneling current (see red dashed and blue dashed-dotted curves in Fig. 9).

Another situation occurs when the system is initially prepared in the symmetric single-electron state. In this case in coupled quantum dots the transitions between singlet two-electron state $S^0$ and symmetric single-electron state *S* are allowed as well as transitions between single-electron state *S* and empty states. Thus, depending on the applied bias voltage one of these channels or both of them can contribute to the tunneling current and zero frequency noise. Tunneling current for $S^0-S-0$ channel is enhanced as well as zero frequency noise in comparison with the simple case of tunneling through the single-electron energy level due to the constructive interference as well as zero frequency noise spectra (see black solid and blue dashed-dotted curves in Figs. 8 and 9). Relative contributions of electron transitions between $S^0$ and *S* states and *S* and empty states to the tunneling current and zero frequency noise are shown in Fig. 10. Tunneling current reaches its maximum for the asymmetric coupling between left and right leads when $\gamma _L/\gamma =\sqrt{2}-1$, so coupling with the filled lead is weaker than with the empty one contrary to the case of tunneling through the $T^0-a$ channel when current maximum is achieved for stronger coupling with the filled lead. Zero frequency current noise demonstrates similar dependence on the coupling asymmetry between the left and right leads of tunneling contact.

**Figure 11:**
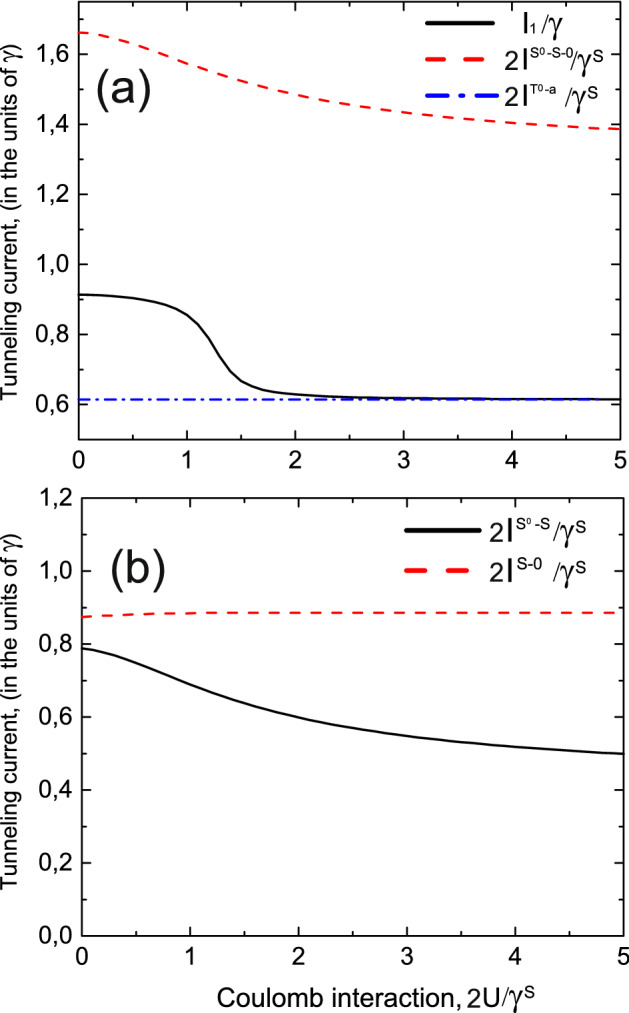
Tunneling current as a function of Coulomb interaction ($2U/\gamma _S$) for a single-level in the quantum dot $I_1/\gamma$ (black curve), for the $S^0-S-0$ channel $2I^{S^0-S-0}/\gamma ^S$ (red dashed curve) and for the $T^0-a$ channel $I^{T^0a}/\gamma ^{T^0a}=2I^{T^0a}/\gamma _{S}$ (blue dashed-dotted curve) for symmetric coupling between the left and right leads. Temperature is equal to $0.01\varepsilon$.

To analyze the role of Coulomb interaction we calculated the dependencies of tunneling current on the value of Coulomb correlations for the symmetric tunneling contact. Tunneling current as a function of Coulomb interaction ($2U/\gamma _S$) for the $S^0-S-0$ channel $2I^{S^0-S-0}/\gamma _S$ (red dashed curve) and for the $T^0-a$ channel $I^{T^0a}/\gamma ^{T^0a}=2I^{T^0a}/\gamma ^{S}$ (blue dashed-dotted curve) is shown in Fig. 11. Currents are compared with the value of tunneling current through single-level quantum dot with Coulomb interaction (see black curve in Fig. 11). Tunneling rate $\gamma ^{T^0a}=\gamma ^S/2$ is independent on Coulomb interaction as well as energies $E_{T^0}$, $\varepsilon _{a}$ and occupation number $N_{T}^{T^0a}$. Thus, tunneling current through the $T^0-a$ channel does not depend on Coulomb interaction. Tunneling current through the $S^0-S-0$ channel is sensitive to the value of Coulomb interaction because tunneling rate $\gamma ^{S^0S}$ depends on Coulomb interaction as well as the singlet state energy $E_{S^0}$ and occupation number $N_{T}^{S^0S}$. For $2U/\gamma ^S \gg 1$ the following ratio between tunneling rates occurs $\gamma ^{S^0S}\simeq \gamma ^S/2$, while for small Coulomb interaction $\gamma ^{S^0S}\simeq \gamma ^S$. Tunneling current through the $S^0-S-0$ channel is the largest one for all the values of Coulomb correlations (see red dashed curve in Fig. 11a). It slightly decreases with the growth of $2U/\gamma ^S$. Figure 11b shows the dependence on Coulomb interaction of relative contributions of electron transitions between $S^0$ and *S* states and *S* and empty states to the tunneling current. Current through the $T^0-a$ channel does not depend on the strength of Coulomb interaction and its amplitude is the smallest one (see blue dashed dotted curve in Fig. 11a). Tunneling current through the single-level quantum dot with Coulomb interaction $I_1/\gamma$ is calculated over the expression (26) in Supplementary Appendix III and is shown by the black curve in Fig. 11a. It demonstrates most pronounced dependence on the value of Coulomb interaction and is suppressed for strong Coulomb interaction in comparison with the situation when Coulomb interaction is absent. For strong Coulomb correlations tunneling current value in this case tends to the value of tunneling current through the $T^0-a$ channel.

We would like to summarize obtained results in a Table 1.

**Table 1:** Currents and noise maximum values as a functions of contact asymmetry.

Contact symmetry	Maximum values of currents	Maximum values of noise
$\gamma _L/\gamma =0.5$	$I_1/\gamma$	$S_1/\gamma$
$\gamma _L/\gamma >0.5$	$I^{S^{0}-S-0}/\gamma ^{S^{0}-S}$	$S^{T^{0}-a}/\gamma ^{T^{0}-a}$
$\gamma _L/\gamma <0.5$	$I^{T^{0}-a}/\gamma ^{T^{0}-a}$	$S^{S^{0}-S-0}/\gamma ^{S^{0}-S}$

## Fano factor

Let us now analyze the dependence of tunneling current, zero frequency noise and Fano factor on the applied bias voltage. In the most simple case of single type of carriers tunneling current dependence on the applied bias voltage has a very simple form^55,56^:

31 $$\begin{aligned} \frac{I_{1}(eV)}{\gamma }=\frac{1}{\pi }\frac{4\gamma _L\gamma _R}{\gamma ^{2}}[\arctan \left( \frac{eV-\varepsilon }{\gamma }\right) -\arctan \left( \frac{-\varepsilon }{\gamma }\right) ] \end{aligned}$$

where tunneling rate $\gamma =\gamma _L+\gamma _R$ is directly the sum of tunneling rates from the quantum dot to the left and right leads of tunneling contact. The zero frequency noise dependence on the applied bias voltage can be also written in the explicit form^57,58^:

32 $$\begin{aligned} \frac{S_{1}^{0}(0,eV)}{\gamma }=\frac{I_{1}(eV)}{\gamma }\left[ 1-\left( \frac{4\gamma _L\gamma _R}{\gamma ^{2}}\right) ^{2}\frac{\Phi (eV)}{I_{1}(eV)/\gamma }\right] , \end{aligned}$$

where function $\Phi (eV)$ has the following form

33 $$\begin{aligned} \Phi (eV)=\frac{1}{2\pi }[\arctan \left( \frac{eV-\varepsilon }{\gamma }\right) -\arctan \left( \frac{-\varepsilon }{\gamma }\right) ] +\frac{1}{2}\left[ \frac{(eV-\varepsilon )/\gamma }{\frac{(eV-\varepsilon )^{2}}{\gamma ^{2}}+1}+\frac{\varepsilon /\gamma }{\frac{\varepsilon ^{2}}{\gamma ^{2}}+1}\right] . \end{aligned}$$

Expressions (31)–(32) can be applied both for the analysis of tunneling current and zero frequency noise spectra in a wide range of system parameters for arbitrary value of applied bias voltage. A well-known Landauer–Büttiker formalism can be used in the limit of small values of applied bias, when the ration $eV/\gamma \ll 1$ occurs. In the limit $\varepsilon /\gamma \ll eV/\gamma \ll 1$ expressions (31)–(32) directly reproduce the Landauer–Büttiker formalism and expressions for tunneling current and zero frequency noise have the following form

34 $$\begin{aligned} \frac{I_{1}(eV)}{\gamma }\simeq \frac{4\gamma _L\gamma _R}{\gamma ^{2}}\frac{eV}{\gamma } \end{aligned}$$

and

35 $$\begin{aligned} \frac{S_{1}^{0}(0,eV)}{\gamma }\simeq \frac{4\gamma _L\gamma _R}{\gamma ^{2}}\left( 1-\frac{4\gamma _L\gamma _R}{\gamma ^{2}}\right) \frac{eV}{\gamma }. \end{aligned}$$

In the limit $eV/\gamma \ll 1 \ll \varepsilon /\gamma$ one can get another expressions for tunneling current and zero frequency noise, which also reproduce the Landauer–Büttiker formalism

36 $$\begin{aligned} \frac{I_{1}(eV)}{\gamma }\simeq \frac{4\gamma _L\gamma _R}{\varepsilon ^{2}+\gamma ^{2}}\frac{eV}{\gamma } \end{aligned}$$

and

37 $$\begin{aligned} \frac{S_{1}^{0}(0,eV)}{\gamma }\simeq \frac{4\gamma _L\gamma _R}{\varepsilon ^{2}+\gamma ^{2}}\left( 1-\frac{4\gamma _L\gamma _R}{\varepsilon ^{2}+\gamma ^{2}}\right) \frac{eV}{\gamma }. \end{aligned}$$

So, the proposed approach directly reproduces the Landauer–Büttiker formalism for $eV/\gamma \ll 1$ and extends it for larger values of applied bias, it gives the possibility to analyze tunneling current and noise spectra even in the limit when $eV/\gamma \gg 1$.

Let us now analyze the I–V characteristics and the dependence of zero frequency noise on the applied bias for the $T^{0}-a$ and $S^{0}-S-0$ channels. Expressions for the tunneling current and zero frequency noise dependencies on the applied bias voltage in the case of tunneling through the $T^0-a$ channel follow from (20), (31) and (21), (32), correspondingly, and have the following form

38 $$\begin{aligned} \frac{I^{T^0a}(eV)}{\gamma ^{T^0a}}=\frac{1}{\pi }\frac{4\gamma _{L}^{T^0a}\gamma _{R}^{T^0a}}{(\gamma ^{T^0a})^{2}[2-N_{T}^{T^0a}(eV)]}\left[ \arctan \left( \frac{eV-E^{T^0a}}{\gamma ^{T^0a}}\right) -\arctan \left( \frac{-E^{T^0a}}{\gamma ^{T^0a}}\right) \right] \end{aligned}$$

and

39 $$\begin{aligned} \frac{S^{T^0a}(eV)}{\gamma ^{T^0a}}=\frac{I^{T^0a}(eV)}{\gamma ^{T^0a}}\left[ 1-\left( \frac{4\gamma _{L}^{T^0a}\gamma _{R}^{T^0a}}{(\gamma ^{T^0a})^{2}}\right) ^{2} \frac{\Phi (eV,E^{T^0a})}{I_{1}^{T^0a}(eV)/\gamma ^{T^0a}}\right] , \end{aligned}$$

where $N_T^{T^{0}a}=\frac{\gamma _{L}^{T^{0}a}\Phi _{k}^{T^{0}a}+\gamma _{R}^{T^{0}a}\Phi _{p}^{T^{0}a}}{\gamma _{L}^{T^{0}a}+\gamma _{R}^{T^{0}a}}$ and $N_T^{T^{0}a}\equiv N_T(E_{T^{0}}-\varepsilon _a)$. Functions $\Phi _{k(p)}^{T^{0}a}\equiv \Phi _{k(p)}(E_{T^{0}}-\varepsilon _a)$ read

40 $$\begin{aligned} \Phi _{k(p)}(E_{T^{0}}-\varepsilon _a)= \frac{1}{2\pi }i\int d\varepsilon _{k(p)}f_{k(p)}(\varepsilon _{k(p)})\times \left[ \frac{1}{E_{T^{0}}-\varepsilon _a+i(\gamma _{L}+\gamma _R)-\varepsilon _{k(p)}}\right.\left. -\frac{1}{E_{T^{0}}-\varepsilon _a-i(\gamma _{L}+\gamma _R)-\varepsilon _{k(p)}}\right] . \end{aligned}$$

**Figure 12:**
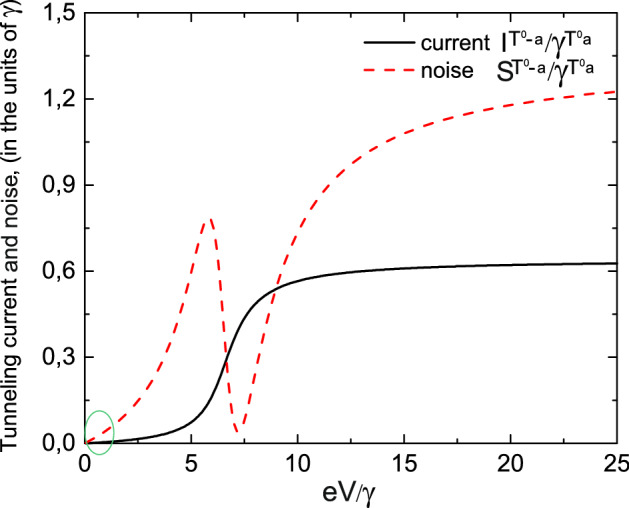
Tunneling current and zero frequency noise as a functions of applied bias for symmetric tunneling contact in the limit $\varepsilon /\gamma \gg 1$ for the $T^{0}-a$ channel. Tunneling current is shown by the solid black line and zero frequency noise is depicted by the red dashed curve. Green circle shows the system parameters where Landauer–Büttiker formalism can be applied. Temperature is equal to $0.01\varepsilon$.

Expression for tunneling current $I_{1}^{T^0a}(eV)$ is given by (31) with the following changes of energy $\varepsilon \rightarrow E^{T^0a}$ and tunneling rate $\gamma \rightarrow \gamma ^{T^0a}$. Function $\Phi (eV,E^{T^0a})$ is determined by expression (33) with the same changes for energy and relaxation rate as for the tunneling current $I_{1}^{T^0a}(eV)$. The dependencies of tunneling current and zero frequency noise on the applied bias are shown in Fig. 12. Tunneling current demonstrates a typical step like dependence on the applied bias. The behavior of zero frequency noise is a non-monotonic one. It first increases with the growth of applied bias, reaches maximum and decreases to the zero value when noise is nearly absent in the system with further monotonic growth. Green circle in Fig. 12 shows the area of system parameters where Landauer–Büttiker formalism can be applied.

**Figure 13:**
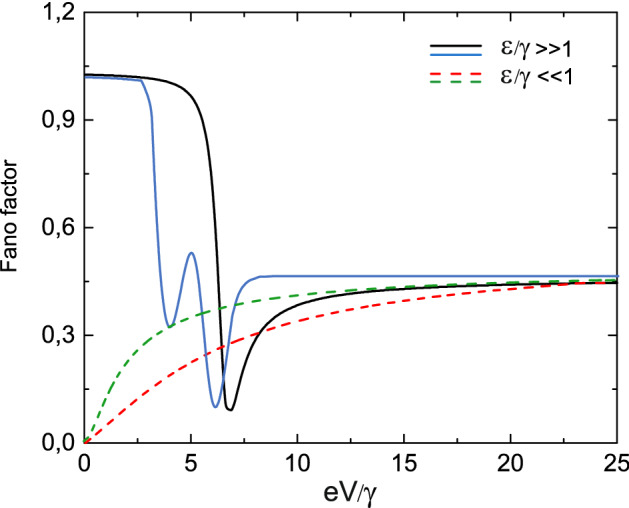
Fano factor as a functions of applied bias for symmetric tunneling contact for both $T^0-a$ and $S^0-S-0$ channels in two limiting cases $\varepsilon /\gamma \gg 1$ and $\varepsilon /\gamma \ll 1$ for the $T^{0}-a$ channel. The limiting case $\varepsilon /\gamma \gg 1$ is shown by the solid lines (black line corresponds to the $T^0-a$ channel and blue line depicts results obtained for the $S^0-S-0$ channel) and the limiting case $\varepsilon /\gamma \ll 1$ is depicted by the dashed curves (black line corresponds to the $T^0-a$ channel and blue line depicts results obtained for the $S^0-S-0$ channel). Temperature is equal to $0.01\varepsilon$.

We also calculated the dependence of Fano factor $F(eV)=\frac{S(0,eV)}{I(eV)}$ both on the energy of the single occupied state ($\varepsilon _1=\varepsilon _2=\varepsilon$) and the applied bias voltage. The dependence of Fano factor on the applied bias voltage for two limiting cases ($\varepsilon /\gamma \gg 1$ and $\varepsilon /\gamma \ll 1$) is shown in Fig. 13 by solid black and dashed red curves correspondingly. In the limit $\varepsilon /\gamma \gg 1$ Fano factor demonstrates non-monotonic behavior. For $\varepsilon /\gamma \gg 1$ the maximum value of Fano factor corresponds to the zero value of applied bias. The growth of applied bias leads to the decreasing of Fano factor, it reaches minimum value and then monotonically increases to 1/2. In the limit $\varepsilon /\gamma \gg 1$ Fano factor reveals monotonic behavior. It is equal to zero for $eV=0$ and it monotonically aspires to 1/2 with the growth of applied bias.

**Figure 14:**
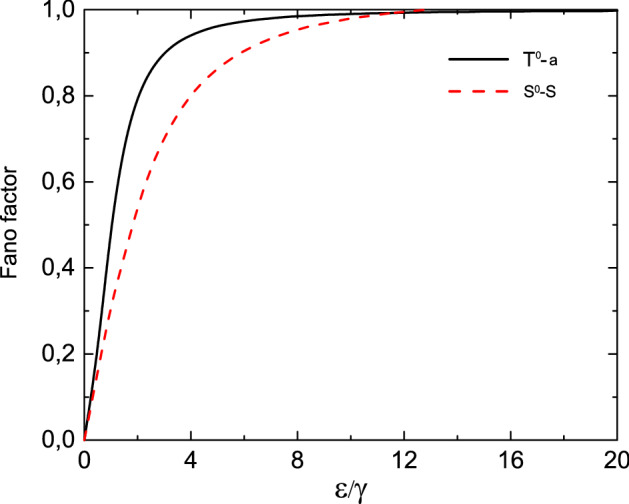
Fano factor as a functions of energy for the symmetric tunneling contact in the limiting case $eV/\gamma \ll 1$ for the $T^{0}-a$ channel—black solid curve and for the $S^{0}-S-0$ channel—red dashed curve. Temperature is equal to $0.01\varepsilon$.

For a fixed value of applied bias in the limit of $eV/\gamma \ll 1$ the dependence of Fano factor on the energy of the single occupied state ($\varepsilon _1=\varepsilon _2=\varepsilon$) also reveals monotonic behavior. It starts from zero value and aspires monotonically to the maximum value equal to unity (see solid black curve in Fig. 14).

Expressions for the tunneling current and zero frequency noise dependencies on the applied bias voltage in the case of tunneling through the $S^0-S-0$ channel follow from (24), (31) and (25), (32), correspondingly and have the following form

41 $$\begin{aligned} \frac{I^{S^0-S-0}(eV)}{\gamma ^{S^0S}}= & {} \frac{I^{S^0-S}(eV)}{\gamma ^{S^0S}}+\frac{I^{S-0}(eV)}{\gamma ^{S^0S}}=\frac{1}{\pi }\frac{4\gamma _{L}^{S^0S}\gamma _{R}^{S^0S}}{(\gamma ^{S^0S})^{2}[1+N_{T}^{S}(eV)+N_{T}^{S^0S}(eV)]}N_{T}^{S}(eV)\nonumber \\&\times \left[ \arctan \left( \frac{eV-E^{S^0S}}{\gamma ^{S^0S}}\right) -\arctan \left( \frac{-E^{S^0S}}{\gamma ^{S^0S}}\right) \right] \nonumber \\&+2\frac{1}{\pi }\frac{4\gamma _{L}^{S^0S}\gamma _{R}^{S^0S}}{(\gamma ^{S^0S})^{2}[1+N_{T}^{S}(eV)+N_{T}^{S^0S}(eV)]}\left[ 1-N_{T}^{S^0S}(eV)\right] \nonumber \\&\times \left[ \arctan \left( \frac{eV-E^{S^0S}}{2\gamma ^{S^0S}}\right) -\arctan \left( \frac{-E^{S^0S}}{2\gamma ^{S^0S}}\right) \right] \end{aligned}$$

and

42 $$\begin{aligned} \frac{S^{S^0-S-0}(0,eV)}{\gamma ^{S^0S}}= & {} \frac{I^{S^0-S}(eV)}{\gamma ^{S^0S}}\left[ 1-\left( \frac{4\gamma _{L}^{S^0S}\gamma _{R}^{S^0S}}{(\gamma ^{S^0S})^{2}}\right) ^{2}\frac{\Phi (eV,E^{S^0S})}{I_{1}^{S^0S}(eV)/\gamma ^{S^0S}}\right] \nonumber \\&+\frac{I^{S-0}(eV)}{\gamma ^{S^0S}}\left[ 1-\left( \frac{4\gamma _{L}^{S^0S}\gamma _{R}^{S^0S}}{(\gamma ^{S^0S})^{2}}\right) ^{2}\frac{\Phi (eV,E^{S})}{I_{1}^{S}(eV)/\gamma ^{S^0S}}\right] \end{aligned}$$

**Figure 15:**
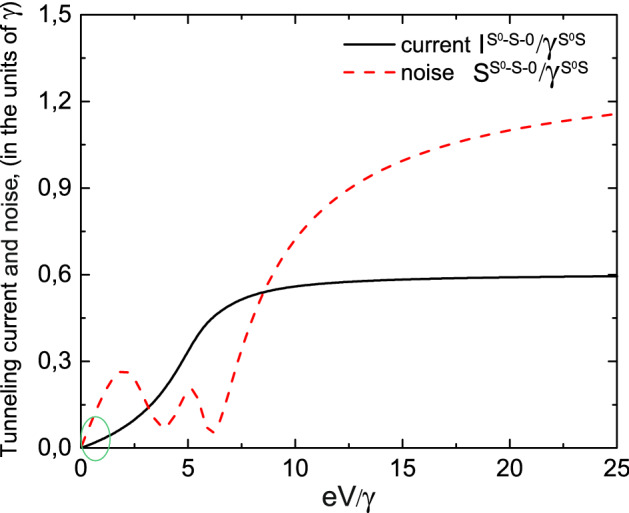
Tunneling current and zero frequency noise as a functions of applied bias for symmetric tunneling contact in the limit $\varepsilon /\gamma \gg 1$ for the $S^{0}-S-0$ channel. Tunneling current is shown by the solid black line and zero frequency noise is depicted by the red dashed curve. Green circle shows the system parameters where Landauer–Büttiker formalism can be applied. Temperature is equal to $0.01\varepsilon$.

Expressions for tunneling current $I_{1}^{S^0-S}(eV)$ and $I_{1}^{S-0}(eV)$ are given by (31) with the corresponding changing of energies $\varepsilon \rightarrow E^{S^0S}$ and $\varepsilon \rightarrow E^{S}$ and tunneling rate $\gamma \rightarrow \gamma ^{S^0S}$. Function $\Phi (eV,E^{S^0S})$ is determined by expression (33) with the same changes for energy and relaxation rate as for the tunneling current $I_{1}^{S-0}(eV)$. The dependencies of the tunneling current and zero frequency noise on the applied bias are shown in Fig. 15. Tunneling current demonstrates a typical step like behavior while the zero frequency noise reveals a non-monotonic behavior. Contrary to the results obtained for the $T^{0}-a$ channel zero frequency noise for the $S^{0}-S$ channel has two local minima and maxima. It first increases with the growth of applied bias, twice reaches maximum and minimum with further monotonic growth. Green circle in Fig. 15 shows the range of system parameters where Landauer–Büttiker formalism can be applied.

The dependence of Fano factor on the applied bias voltage for two limiting cases ($\varepsilon /\gamma \gg 1$ and $\varepsilon /\gamma \ll 1$) is shown in Fig. 13 by solid blue and dashed green lines. In the limit $\varepsilon /\gamma \gg 1$ Fano factor demonstrates nonmonotonic behavior. For $\varepsilon /\gamma \gg 1$ the maximum value of Fano factor corresponds to the zero value of applied bias. The growth of applied bias leads to the decreasing of Fano factor, it twice reaches local minima and local maxima and then monotonically increases to 1/2. In the limit $\varepsilon /\gamma \gg 1$ Fano factor reveals monotonic behavior. It has zero value for $eV=0$ and monotonically aspires to 1/2 with the growth of applied bias. It is clearly evident that in the regime when $eV/\gamma \ll 1$ Fano factor for the $T^0-a$ channel is smaller than for the $S^0-S-0$ channel, while in the situation when $\varepsilon /\gamma \gg 1$ there exist parameters ranges when Fano factor for the $T^0-a$ channel is larger than for the $S^0-S-0$ channel.

For the fixed value of applied bias in the limit of $eV/\gamma \ll 1$ the dependence of Fano factor on the energy of single-electron state ($\varepsilon _1=\varepsilon _2=\varepsilon$) was also analyzed. It starts from zero value and aspires monotonically to its maximum value equal to unity (see solid black curve in Fig. 14). It is necessary to mention that in a considered parameters range Fano factor for the $T^0-a$ channel is smaller than for the $S^0-S-0$ channel.

## Conclusions

A general approach for analyzing tunneling current and its zero frequency noise in various systems where electron transport occurs through the intermediate structure with localized electrons was developed. The application of suggested approach allows to analyze electron transport through multi-electron states with Coulomb correlations beyond mean-field approximation and opens the possibility to study the influence of spatial and spin symmetry of the total system on the tunneling characteristics. The proposed approach is based on Keldysh diagram technique in pseudo-particle representation with operator constraint on the total number of pseudo-particles. It was shown that for the system of two correlated quantum dots each coupled to both leads, tunneling current and its zero frequency noise strongly depend on initial system state, which determines the path of allowed transitions between multi-electron states in quantum dots—the tunneling channel. If tunneling current flows through the entangled triplet state with zero total spin projection on the z axis both current and zero frequency noise are suppressed. Contrary, if the tunneling current flows through the singlet two-electron state and symmetric single electron state, both tunneling current and zero frequency noise are enhanced in comparison with electron transport through single uncorrelated localized electron state. We also revealed that the maximum value of tunneling current is achieved for the asymmetric coupling between the left and right leads. If triplet two-electron state is involved in the tunneling process current and zero frequency noise maximum values are achieved for stronger coupling with the filled lead, while for the singlet state current and zero frequency noise maximum values correspond to the stronger coupling with the empty lead. It was also found that tunneling current through the triplet two-electron—antisymmetric single-electron state does not depend on the value of Coulomb interaction. Tunneling current trough the singlet two-electron—symmetric single-electron state decreases with the growth of Coulomb interaction. The obtained nonmonotonic behavior of Fano factor as a function of applied bias reveals the possibility to control the noise to signal ration in correlated quantum dots.

## Supplementary Information

Supplementary Information.
