# Supplementary material for: Tunneling current and noise of entangled electrons in correlated double quantum dot
Source: Sci Rep. 2021 Apr 29;11:9336. doi: 10.1038/s41598-021-88721-7 (PMC8085215; doi:10.1038/s41598-021-88721-7)
Supplement: Supplementary file 1 — Supplementary Information. [file 41598_2021_88721_MOESM1_ESM.pdf]

Supplementary materials: Tunneling current and noise of entangled electrons in correlated double quantum dot

N. S. Maslova[1,+], P. I. Arseyev[2,+], and V. N. Mantsevich[3,*,+]

[1]Quantum Technology Center and Quantum electronics department, Faculty of Physics, Lomonosov Moscow State University, 119991 Moscow, Russia
[2]P.N. Lebedev Physical Institute RAS, 119991 Moscow, Russia
[3]Quantum Technology Center and department of Semiconductor physics and Cryoelectronics, Faculty of Physics, Lomonosov Moscow State University, 119991 Moscow, Russia

ABSTRACT

Below in **Appendix I**, **Appendix II** and **Appendix III** parts some detailed calculations are performed

1 Appendix I

One can derive kinetic equations for the pseudo-particle occupation numbers $N_i^{\sigma}=\langle\hat{f}_{i\sigma}^{\dagger}\hat{f}_{i\sigma}\rangle$, $N_j^{\sigma-\sigma}=\langle\hat{d}_j^{\dagger\sigma-\sigma}\hat{d}_j^{\sigma-\sigma}\rangle$ and $N_b=\langle\hat{b}^{\dagger}\hat{b}\rangle$ by averaging equations of motion for the operators and by decoupling the electrons occupation numbers in quantum dots from the reservoir occupation numbers. Such decoupling procedure is reasonable provided that Kondo correlations could be neglected when applied bias eV strongly exceeds all the tunneling rates. So, after taking into account the constraint on possible physical states, the following non-stationary system of equations can be obtained for the pseudo-particle occupation numbers[1,2]:

$$\begin{aligned}
\frac{\partial N_j^{\sigma-\sigma}}{\partial t} &= -\sum_{i\sigma}[\lambda_{jiL}^{\sigma-\sigma}(1-\Phi_{k-\sigma}^{ji})N_j^{\sigma-\sigma}-\lambda_{jiL}^{\sigma-\sigma}\Phi_{k-\sigma}^{ji}N_i^{\sigma}]-\sum_{i\sigma}[\lambda_{jiR}^{\sigma-\sigma}(1-\Phi_{p-\sigma}^{ji})N_j^{\sigma-\sigma}-\lambda_{jiR}^{\sigma-\sigma}\Phi_{p-\sigma}^{ji}N_i^{\sigma}],\\
\frac{\partial N_i^{\sigma}}{\partial t} &= \sum_j\lambda_{jiL}^{\sigma-\sigma}(1-\Phi_{k-\sigma}^{ji})N_j^{\sigma-\sigma}-\sum_j[\lambda_{jiL}^{\sigma-\sigma}\Phi_{k-\sigma}^{ji}N_i^{\sigma}-\lambda_{iL}(1-\Phi_{k\sigma}^{i})N_i^{\sigma}+\lambda_{iL}\Phi_{k\sigma}^{i}N_b]\\
&+ \sum_j\lambda_{jiR}^{\sigma-\sigma}(1-\Phi_{p-\sigma}^{ji})N_j^{\sigma-\sigma}-\sum_j[\lambda_{jiR}^{\sigma-\sigma}\Phi_{p-\sigma}^{ji}N_i^{\sigma}-\lambda_{iR}(1-\Phi_{p\sigma}^{i})N_i^{\sigma}+\lambda_{iR}\Phi_{p\sigma}^{i}N_b],\\
\frac{\partial N_b}{\partial t} &= \sum_{i\sigma}\lambda_{iL}[N_i^{\sigma}(1-\Phi_{k\sigma}^{i})-\Phi_{k\sigma}^{i}N_b]+\sum_{i\sigma}\lambda_{iR}[N_i^{\sigma}(1-\Phi_{p\sigma}^{i})-\Phi_{p\sigma}^{i}N_b],\\
\frac{\partial N_j^{\sigma\sigma}}{\partial t} &= -\sum_i[\lambda_{jiL}^{\sigma\sigma}(1-\Phi_{k\sigma}^{ji}),
\end{aligned}\qquad(1)$$

where kinetic coefficients are

$$\begin{aligned}
\lambda_{iL(R)} &= \lambda_{jiL(R)}^{\sigma\sigma}=2\gamma_{L(R)}|\mu_i+\nu_i|^2,\\
\lambda_{jiL(R)}^{\sigma-\sigma} &= 2\gamma_{L(R)}|\alpha_j\mu_i+\beta_j\nu_i+\delta_j\nu_i+\gamma_j\mu_i|^2.
\end{aligned}\qquad(2)$$

Index $i=a,S$ and the relaxation rate $\gamma_{L(R)}=\pi\nu_0 t_{L(R)}^2$ (ν_0 is the electronic density of states in the reservoir). Functions $\Phi_{k-\sigma}^{ji}$ and $\Phi_{k\sigma}^{i}$ depend on reservoir properties and have the form:

$$\begin{aligned}
\Phi_{k-\sigma}^{ji} &= \frac{1}{2\pi}i\int d\varepsilon_k f_k^{\sigma}(\varepsilon_k)\times[\frac{1}{E_j^{\sigma\sigma'}-\varepsilon_i+i\gamma_{jiL}-\varepsilon_k}-\frac{1}{E_j^{\sigma\sigma'}-\varepsilon_i-i\gamma_{jiL}-\varepsilon_k}],\\
\Phi_{k\sigma}^{i} &= \frac{1}{2\pi}i\int d\varepsilon_k f_k^{\sigma}(\varepsilon_k)\times[\frac{1}{\varepsilon_i+i\gamma_{iL}-\varepsilon_k}-\frac{1}{\varepsilon_i-i\gamma_{iL}-\varepsilon_k}],
\end{aligned}\qquad(3)$$

where

$$\gamma_{iL} = \lambda_i/2,$$
$$\gamma_{jiL} = \lambda_{ji}^{\sigma-\sigma}/2 \quad (4)$$

and $f_k^{\sigma}(\varepsilon_k)$ - is the Fermi distribution function of the electrons in the reservoir. Similar expressions are valid for the right reservoir with the following indexes substitution $L \leftrightarrow R$ and $k \leftrightarrow p$.

System of kinetic equations for pseudo-particles (1) has to be solved with the initial conditions for each pseudo-particle occupation number. In case of symmetric coupling to the reservoir, the system of equations can be solved as two independent systems of equations. One of them contains only the equations for the occupation numbers N^{T_0} and $N^{a\pm}$ and the other one describes the dynamics of the occupation numbers $N^{T\pm}$, N^{S_0}, $N^{S\pm}$, and N_b. It occurs due to the selection rules coming from the symmetry properties of the system, $\gamma^a = \gamma^{T_0 s} = \gamma^{S_0 a} = 0$. So, there is no mixing between the triplet T^0 and singlet channels $a_{\pm}$, consequently contribution to the tunneling current and noise spectra from these channels can be calculated separately. So it is also reasonable to group the initial conditions for each system and determine them as $N_I(0)$ and $N_{II}(0)$.Due to the constraint on possible physical states one has $N_I(0) + N_{II}(0) = 1$. Consequently, the corresponding initial conditions are

$$N_I(0) = N^{T^0}(0) + 2N^{a^{\pm}}(0),$$
$$N_{II}(0) = N^{S^0}(0) + 2N^{S^{\pm}}(0) + N_b(0). \quad (5)$$

For initial antisymmetric state $N_I(0) = 1$ and $N_{II}(0) = 0$ and for initial symmetric state $N_I(0) = 0$ and $N_{II}(0) = 1$.

The stationary values of partial pseudo-particles occupation numbers are:

$$N^{S^0 st} = \frac{N_{II}(0) N_{T\sigma}^{S} N_{T-\sigma}^{S^0 S}}{1 + N_T^S - N_T^{S_0 S}},$$
$$N^{S^{\pm} st} = \frac{N_{II}(0) N_{T\sigma}^{S}(1 - N_{T-\sigma}^{S^0 S})}{1 + N_T^S - N_T^{S_0 S}},$$
$$N_b^{st} = \frac{N_{II}(0)(1 - N_{T\sigma}^{S})(1 - N_{T-\sigma}^{S^0 S})}{1 + N_T^S - N_T^{S_0 S}} = b,$$
$$N^{T^0 st} = \frac{N_I(0) N_{T-\sigma}^{T^0 a}}{2 - N_{T-\sigma}^{T^0 a}},$$
$$N^{a^{\pm} st} = \frac{N_I(0)(1 - N_{k-\sigma}^{T^0 a})}{2 - N_{T-\sigma}^{T^0 a}}, \quad (6)$$

with $N_T^{T^0 a} = \frac{\gamma_L^{T^0 a}\Phi_k^{T^0 a} + \gamma_R^{T^0 a}\Phi_p^{T^0 a}}{\gamma_L^{T^0 a} + \gamma_R^{T^0 a}}$, $N_T^{S^0 S} = \frac{\gamma_L^{S^0 S}\Phi_k^{S^0 S} + \gamma_R^{S^0 S}\Phi_p^{S^0 S}}{\gamma_L^{S^0 S} + \gamma_R^{S^0 S}}$ and $N_T^S = \frac{\gamma_L^S \Phi_k^S + \gamma_R^S \Phi_p^S}{\gamma_L^S + \gamma_R^S}$, where $N_T^{T^0 a} \equiv N_T(E_{T^0} - \varepsilon_a)$, $N_T^{S^0 S} \equiv N_T(E_{S^0} - \varepsilon_S)$ and $N_T^S \equiv N_T(\varepsilon_S)$.

2 Appendix II

2.1 Current in the absence of Coulomb interaction: single type of carriers and two different types of carriers

To understand the suggested approach we will analyze a well known simple model with single level quantum dot localized between the leads of the tunneling contact in the absence of Coulomb repulsion. In this case one should retain only index $l = 1$ in the first term in $\hat{H}_{dot}$ and in $\hat{H}_{tun}$ parts of the system Hamiltonian (2), (4). All other terms in $\hat{H}_{dot}$ part of the Hamiltonian should be omitted. In this case creation of localized electron with energy ε is given by the creation operator $\hat{c}^{\dagger} = \hat{f}^{\dagger}\hat{b}$

Diagrams contributing to the current are shown in Fig.1a. For single type of carriers tunneling current reads:

$$I_1 = \gamma_L \int d\omega d\omega_1 [N^R(\omega) J_k^<(\omega_1) B^<(\omega - \omega_1) + N^<(\omega) J_k^>(\omega - \omega_1) B^A(\omega - \omega_1)$$
$$- B^<(\omega - \omega_1) J_k^<(\omega_1) N^A(\omega) - B^R(\omega - \omega_1) J_k^>(\omega - \omega_1) N^<(\omega)], \quad (7)$$

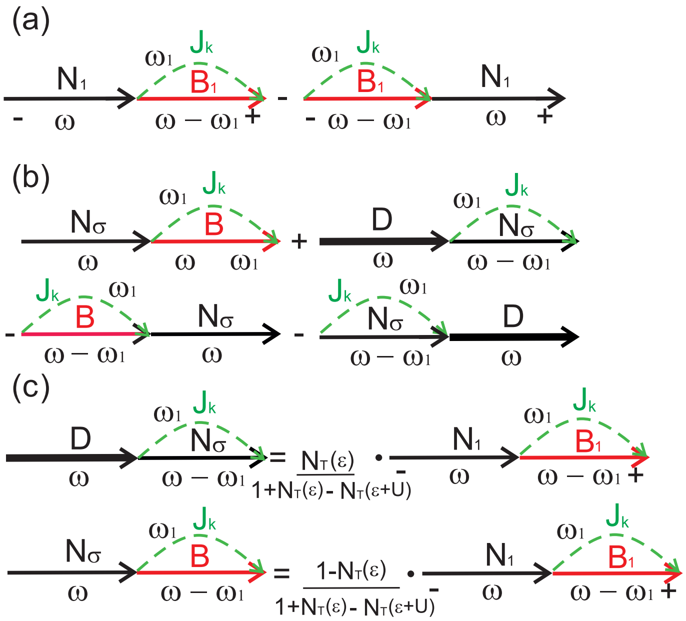

Figure 1. (Color online) Leading diagrams contributing to the tunneling current single level quantum dot a) for single type of carriers; b)-c) for two types of carriers in the presence of Coulomb interaction. Panel c) demonstrates the link between different contributions to the tunneling current in the presence of Coulomb interaction with tunneling current for single type of carriers without Coulomb interaction.

with $\gamma_{L(R)} = \pi \nu_0 t^2_{L(R)}$ and ν_0 being an unperturbed density of states in the leads. Here for real electrons in the leads with a given spin projection one can use the relations

$$
\begin{aligned}
J_k^{<(>)}(\omega) &= \int d\varepsilon_k G_k^{<(>)}(\omega, k), \\
G_k^{<}(\omega) &= f_k(\omega)\left[G_k^A(\omega) - G_k^R(\omega)\right], \\
G_k^{>}(\omega) &= (f_k(\omega) - 1)\left[G_k^A(\omega) - G_k^R(\omega)\right].
\end{aligned} \tag{8}
$$

Retarded and advanced pseudo-particle Green's functions are defined as

$$
\begin{aligned}
N^{R(A)}(\omega) &= \frac{1}{\omega - \varepsilon \pm i\gamma}, \\
B^{R(A)}(\omega) &= \frac{1}{\omega \pm i\gamma}.
\end{aligned} \tag{9}
$$

Lesser functions are determined in the following way

$$
\begin{aligned}
N^{<}(\omega) &= n\left[N^A(\omega) - N^R(\omega)\right], \\
B^{<}(\omega) &= -b\left[B^A(\omega) - B^R(\omega)\right].
\end{aligned} \tag{10}
$$

Pseudo-particle occupation numbers can be found from kinetic equations with the operator constraint on pseudo-particles numbers $n = N_T(\varepsilon)$ and $b = 1 - N_T(\varepsilon)$ with $N_T(\varepsilon) = \frac{\gamma_L \Phi_k(\varepsilon) + \gamma_R \Phi_p(\varepsilon)}{\gamma_L + \gamma_R}$.

$$
\Phi_{k(p)}(\varepsilon) = \frac{1}{2\pi} i \int d\varepsilon_{k(p)} f_{k(p)}(\varepsilon_{k(p)}) \times \left[\frac{1}{\varepsilon + i(\gamma_L + \gamma_R) - \varepsilon_{k(p)}} - \frac{1}{\varepsilon - i(\gamma_L + \gamma_R) - \varepsilon_{k(p)}}\right].
$$

For the $f_k(\varepsilon_k)$ function E_F is shifted on the eV compared to $f_p(\varepsilon_p)$. Finally, for $|eV|/\gamma_{L(R)} >> 1$

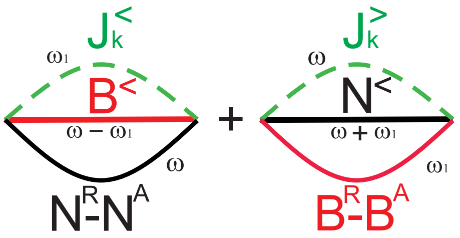

Figure 2. (Color online) Leading diagrams contributing to the zero frequency noise for a single type of carriers.

$$I_1 = 4\frac{\gamma_L\gamma_R}{\gamma_L+\gamma_R}\left[N_k(\varepsilon)(1-N_p(\varepsilon))-N_p(\varepsilon)(1-N_k(\varepsilon))\right]. \tag{11}$$

In the presence of two types of particles (electrons with different spin projections), without Coulomb interaction tunneling channels for each spin are independent. So, tunneling current simply becomes two times larger.

One can also calculate tunneling current and zero frequency noise substituting electronic operators through the pseudo-particle operators $\hat{c}^\dagger_{1\sigma} = \hat{f}^\dagger_\sigma\hat{b} + \hat{d}^\dagger\hat{f}_{-\sigma}$. Operator $\hat{f}^\dagger_{\pm\sigma}$ corresponds to creation of single-occupied electronic state with spin $\pm\sigma$ and operator $d^\dagger$ describes creation of double-occupied electronic state with energy 2ε with corresponding operator constraint on the total number of pseudo-particles $\hat{d}^\dagger\hat{d} + \sum_\sigma \hat{f}^\dagger_\sigma\hat{f}_\sigma + \hat{b}^\dagger\hat{b} = 1$.

Tunneling current in such pseudo-particle representation with $U=0$ can be obtained from diagrams shown in Fig.1b. These diagrams now contain $D^{R(A)}(\omega)$ and $D^<(\omega)$ functions, which read

$$\begin{aligned} D^{R(A)}(\omega) &= \frac{1}{\omega-2\varepsilon\pm i\gamma}, \\ D^<(\omega) &= -d^{\sigma-\sigma}\left[D^A(\omega)-D^R(\omega)\right]. \end{aligned} \tag{12}$$

In this case one can get pseudo-particle occupation numbers from the stationary solution of kinetic equations for pseudo-particles occupation numbers $b = [1-N_T(\varepsilon)]^2$, $n = N_T(\varepsilon)[1-N_T(\varepsilon)]$ and $d = N_T^2(\varepsilon)$ using (1) in **Appendix I**. To obtain analytical expression for diagrams with $D-N_\sigma$ pseudo-particles Green's functions one should multiply expressions obtained for the single type of particles by $N_T(\varepsilon)$ with the substitution $\tilde{\omega} = \omega-\varepsilon$ and for diagrams with $N_\sigma - B$ pseudo-particles Green's functions by multiplying expression for single type of particles by $1-N_T(\varepsilon)$ correspondingly (see Fig.1c with $U=0$). For two different types of carriers $I_T = \sum_\sigma I_T^\sigma = 2I_1$, where $I_T^\sigma = I_1$ is the tunneling current for each spin channel.

2.2 Noise in the absence of Coulomb interaction: single type of carriers and two different types of carriers

One can also obtain expression for the leading term of the noise spectra. Leading diagrams contributing to the zero frequency noise in the absence of Coulomb correlations are shown in Fig.2. The first diagram gives the following contribution to the zero frequency noise in the absence of Coulomb correlations

$$S^0_{B1}(0) = \int d\omega d\omega_1 J_k^<(\omega_1)b(\varepsilon)\left[B^A(\omega-\omega_1)-B^R(\omega-\omega_1)\right]\left[N^R(\omega)-N^A(\omega)\right] = 4\gamma_L\Phi_k(\varepsilon)\left[1-N_T(\varepsilon)\right], \tag{13}$$

where $\gamma_B = \gamma N_T$ and $\gamma_N = \gamma(1-N_T)$. Analogously, contribution from the second diagram reads

$$S^0_{N1}(0) = 4\gamma_L N_T(\varepsilon)\left[1-\Phi_k(\varepsilon)\right]. \tag{14}$$

Taking into account that in the low temperature limit ($T\to 0$) $\Phi_k(\varepsilon)[1-\Phi_k(\varepsilon)]\to 0$, the main contribution to the noise spectrum for $\omega=0$ reads

$$S^0_1(0) = S^0_{B1}(0) + S^0_{N1}(0) = eI_1 = \frac{4\gamma_L\gamma_R}{\gamma}\left[\Phi_k(\varepsilon)(1-\Phi_p(\varepsilon))+\Phi_p(\varepsilon)(1-\Phi_k(\varepsilon))\right]. \tag{15}$$

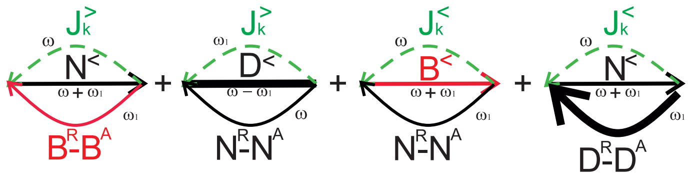

Figure 3. (Color online) Leading diagrams contributing to the zero frequency noise in the presence of Coulomb correlations for a two types of carriers.

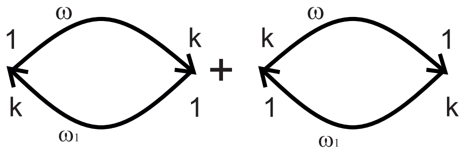

Figure 4. (Color online) Diagrams contributing to the quantum corrections to the tunneling current zero frequency noise for single type of carriers in usual Keldysh diagram technique.

In the absence of Coulomb correlations for the two types of particles main contribution to the tunneling current noise is determined by diagrams shown in Fig.3. As it was mentioned above the analytical expression for leading terms of current noise for diagrams $D-N_\sigma$ and $N_\sigma - B$ pseudo-particle Green's functions are just the same as for the case of tunneling through the single electron energy level. The only difference is that the first type of diagrams should be multiplied by $N_T(\varepsilon)$ and the second one by $1-N_T(\varepsilon)$. Thus,

$$S_1^{0\sigma}(0) = eI_1^\sigma \tag{16}$$

2.3 Noise quantum corrections

In standard Keldysh diagram technique in the case of one type of carriers without Coulomb correlations ($U=0$) one should use diagrams shown in Fig.4 for quantum corrections.

For $|eV| >> \gamma$ and $|\varepsilon| >> \gamma$

$$S_\sigma^1(0) = -2eI_1^\sigma(0)\frac{\gamma_L\gamma_R}{\gamma^2}. \tag{17}$$

For spinless particles in pseudo-particle representation there are no ladder diagrams and quantum corrections to noise are determined by the sum of maximally crossing diagrams (see Fig.5).

The Bethe-Salpeter equation for calculation of C, which is shown in Fig.6 has the following analytical form

$$C(\tilde{\omega},\tilde{\omega}',\omega_3,\omega_4) = C^0(\tilde{\omega},\tilde{\omega}',\omega_3,\omega_4) + \int C^0(\tilde{\omega},\tilde{\omega}',\omega_1,\omega_2)N^A(\tilde{\omega}+\Delta\omega_2)B^R(\tilde{\omega}'-\Delta\omega_2)C(\tilde{\omega}+\Delta\omega_2,\tilde{\omega}'-\Delta\omega_2,\omega_3,\omega_4)d\omega_1 d\omega_2, \tag{18}$$

where $\Delta\omega_2 = \omega_2 - \omega_1$ and $\Delta\omega_3 = \omega_3 - \omega_4$.

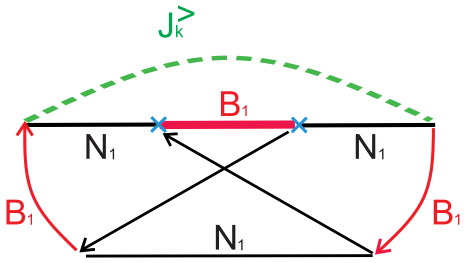

Figure 5. (Color online) Maximally crossing diagrams contributing to the zero frequency noise quantum corrections for single type of carriers.

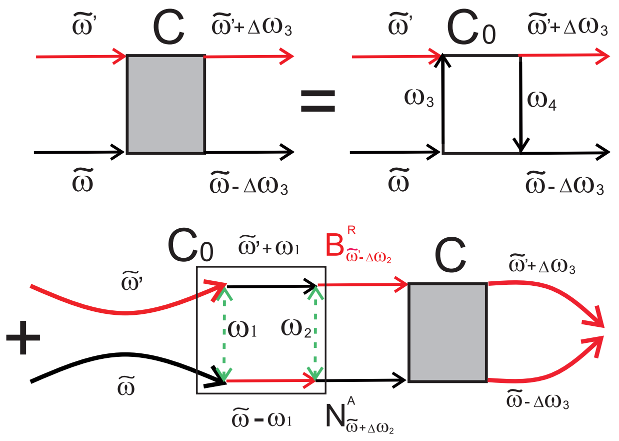

Figure 6. (Color online) The Bethe-Salpeter equation for calculation of maximally crossing diagrams.

Expression for the zero order diagrams containing maximally crossing diagrams C_0 reads:

$$C^0(\omega_1,\omega_2,\tilde{\omega},\tilde{\omega}^{'}) = \gamma^2 J_T^{>}(\omega_1) J_T^{<}(\omega_2) B^A(\tilde{\omega}-\omega_1) N^R(\tilde{\omega}^{'}+\omega_1) \tag{19}$$

with

$$J_T^i(\omega) = \frac{\gamma_L J_k^i(\omega) + \gamma_R J_p^i(\omega)}{\gamma_L+\gamma_R}, \tag{20}$$

where index $i =<,>$ and functions $N^{A(R)}$, $B^{A(R)}$, $N^{<}$ and $B^{<}$ are given by expressions (9)-(10). Quantum corrections to zero frequency noise with $J_k^{>}$ and $N^{<}$ can be written as

$$\begin{aligned} S_{>}^1(0) &= n\gamma_L \int [N^A(\omega+\omega^{'}) - N^R(\omega+\omega^{'})][B^R(\omega+\omega_2-\omega_1) - B^A(\omega+\omega_2-\omega_1)] \\ & N^A(\omega+\omega^{'}+\omega_2-\omega_1) B^R(\omega) C(\omega,\omega^{'},\omega_1,\omega_2) J_k^{>}(\omega^{'}) d\omega^{'} d\omega d\omega_1 d\omega_2. \end{aligned} \tag{21}$$

Expression (21) be evaluated in the limit $|eV|/\gamma >> 1$ and it gives the following contribution to noise quantum corrections

$$S_{>}^1(0) \sim -2\frac{4\gamma_L^2\gamma_R^2}{\gamma^3}\left[1-\Phi_k(\varepsilon)\right]^2 \Phi_p^2(\varepsilon). \tag{22}$$

This expression was obtained taking into account that $\Phi_{k(p)}(\varepsilon)[1-\Phi_{k(p)}(\varepsilon)] \to 0$ for $T \to 0$. Analogously contribution for noise corrections with Green's function $J_k^{<}$

$$S_{<}^1(0) \sim -2\frac{4\gamma_L^2\gamma_R^2}{\gamma^3}\left[1-\Phi_p(\varepsilon)\right]^2 \Phi_k^2(\varepsilon). \tag{23}$$

In the limit of low temperature $T \to 0$ for $eV >> \gamma$ the relation $(1-\Phi_k)^2\Phi_p^2 + \Phi_k^2(1-\Phi_p^2) \sim (\Phi_p - \Phi_k)^2 \sim 1$ takes place. Taking it into account, one can get total expression for zero frequency noise

$$S_1(0) = \frac{4\gamma_L\gamma_R}{\gamma}\left[1 - \frac{2\gamma_L\gamma_R}{\gamma^2}\right]. \tag{24}$$

Performed calculation procedure can be simply generalized for the situation when there exist two types of particles. Fixing the sign of spin index σ for the leads Green function $J_{k\sigma}^{<}(\omega)$ automatically determines the signs of spin index σ for all pseudo-particle Green's functions $N_{\pm\sigma}$ and electron Green's functions in the leads $J_{\sigma}^{T<(>)}$ for maximally crossing diagrams. Thus for two types of particles with $U=0$ $S(0) = 2S_1(0)$.

3 Appendix III

3.1 Tunneling current in the presence of Coulomb correlations

Diagrams contributing to the tunneling current in the presence of Coulomb correlations in the non-crossing approximation are shown in Fig.1b. Pseudo-particle occupation numbers n, d, b should be modified in comparison with the case, when $U=0$. In non-crossing approximation n, d, b can be found from kinetic equations (see **Appendix I**).

$$\begin{aligned} b &= \frac{(1-N_T(\varepsilon))[1-N_T(\varepsilon+U)]}{1+N_T(\varepsilon)-N_T(\varepsilon+U)}, \\ d &= \frac{N_T(\varepsilon)N_T(\varepsilon+U)}{1+N_T(\varepsilon)-N_T(\varepsilon+U)}, \\ n_\sigma = n_{-\sigma} &= \frac{N_T(\varepsilon)[1-N_T(\varepsilon+U)]}{1+N_T(\varepsilon)-N_T(\varepsilon+U)}. \end{aligned} \tag{25}$$

Sum of diagrams with $D-N_\sigma$ pseudo-particle Green's functions is directly the expression for tunneling current in the case of one type of carriers $I_1(\tilde{\varepsilon})$ multiplied by $N_T(\varepsilon)/[1+N_T(\varepsilon)-N_T(\varepsilon+U)]$ with substitution $\tilde{\omega}\to\omega-\varepsilon$ and $\tilde{\varepsilon}\to\varepsilon+U$ and contribution from diagrams with $N_\sigma-B$ pseudo-particles Green's functions diagrams gives $I_{1\sigma}(\varepsilon)$ multiplied by $(1-N_T(\varepsilon+U))/[1+N_T(\varepsilon)-N_T(\varepsilon+U)]$ (see Fig.2c). Finally,

$$I_T^\sigma = \frac{4\gamma_L\gamma_R}{[1+N_T(\varepsilon)-N_T(\varepsilon+U)]\gamma}\times([1-N_T(\varepsilon+U)][\Phi_k(\varepsilon)-\Phi_p(\varepsilon)]+T(\varepsilon)[\Phi_k(\varepsilon+U)-\Phi_p(\varepsilon+U)]). \tag{26}$$

For $0<|\varepsilon|,|\varepsilon+U|<eV$ and $|\varepsilon/\gamma|,|(\varepsilon+U)/\gamma|>>1$, $I_T^\sigma=I_{U=0}^\sigma=I_1$. For $0<\varepsilon<eV$, $\varepsilon+U>eV$ and $\varepsilon<0$, $0<\varepsilon+U<eV$ tunneling current is suppressed due to Coulomb correlations.

3.2 Noise spectra in the presence of Coulomb correlations

Result obtained above gives the possibility to get the answer for the two types of carrier using non-crossing approximation in the presence of Coulomb correlations. The leading contribution to the noise spectra for non-zero U are depicted in Fig.3. Sum of diagrams with $D-N_\sigma$ pseudo-particle Green's functions is directly the expression for leading term of zero frequency noise $S_1^0(0)$ in the case of one type of carriers multiplied by $N_T(\varepsilon)/[1+N_T(\varepsilon)-N_T(\varepsilon+U)]$ with substitution $\tilde{\omega}\to\omega-\varepsilon$ and $\tilde{\varepsilon}\to\varepsilon+U$ and contribution from diagrams with $N_\sigma-B$ pseudo-particles Green's functions diagrams gives $S_1^0(0)$ multiplied by $(1-N_T(\varepsilon+U))/[1+N_T(\varepsilon)-N_T(\varepsilon+U)]$. Thus,

$$S_{\sigma U}^0(0)=eI_T^\sigma, \tag{27}$$

where I_T^σ is determined by expression (26).

For $U\neq 0$ quantum corrections for two types of particles in non-crossing approximation are described by two types of maximally crossing diagrams with $D-N_\sigma$ and $N_\sigma-B$ pseudo-particle Green's function (see Fig.7). As it was mentioned above the spin sign σ of pseudo-fermion Green's functions and the electron Green's function in the leads $J_\sigma^{T<(>)}$ is uniquely determined by the spin sign of external leads electrons Green's function $J_{k\sigma}^{<(>)}$. Zero frequency noise corrections coming from diagrams containing maximally crossing diagrams with $D-N_\sigma$ pseudo-particle Green's functions with particular sign of σ

$$S_{D\sigma}^1(0)=-\frac{N_T(\varepsilon)}{1+N_T(\varepsilon)-N_T(\varepsilon+U)}\frac{4\gamma_L\gamma_R}{\gamma}\frac{2\gamma_L\gamma_R}{\gamma^2}\left[(1-\Phi_k(\varepsilon+U))^2\Phi_p((\varepsilon+U))^2+k\leftrightarrow p\right]. \tag{28}$$

Corrections to the zero frequency noise coming from maximally crossing diagrams with $N_\sigma-B$ pseudo particle Green's functions have the form

$$S_{N\sigma}^1(0)=-\frac{1-N_T(\varepsilon+U)}{1+N_T(\varepsilon)-N_T(\varepsilon+U)}\frac{4\gamma_L\gamma_R}{\gamma}\frac{2\gamma_L\gamma_R}{\gamma^2}. \tag{29}$$

Finally, total zero frequency noise reads

$$\begin{aligned} S_U(0) &= 2\frac{4\gamma_L\gamma_R}{(1+N_T(\varepsilon)-N_T(\varepsilon+U))\gamma}\times[(1-N_T(\varepsilon+U))(1-\frac{2\gamma_L\gamma_R}{\gamma^2})(\Phi_k(\varepsilon)^2(1-\Phi_p(\varepsilon)^2) \\ &+ N_T(\varepsilon)(1-\frac{2\gamma_L\gamma_R}{\gamma^2})(\Phi_k(\varepsilon+U)^2(1-\Phi_p(\varepsilon+U)^2)+k\leftrightarrow p)]. \end{aligned} \tag{30}$$

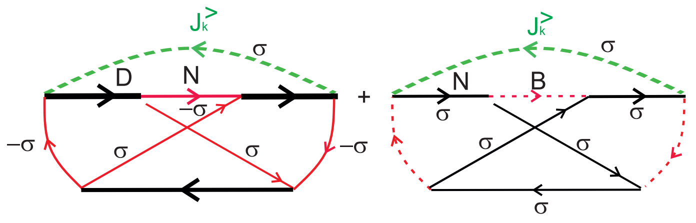

Figure 7. (Color online) Maximally crossing diagrams for zero frequency noise quantum corrections for two types of particles in the presence of Coulomb interaction.

3.3 Tunneling current and zero frequency noise in two quantum dots

For tunneling through $T^0 - a$ channel one can calculate tunneling current and the zero frequency noise in the limit $eV/\gamma >> 1$ using the same diagrams as for $D - N_\sigma$ pseudo-particle Green's functions for $U \neq 0$ with the following changing: all retarded, advanced and lesser Green's functions D should be replaced by the corresponding pseudo-particle Green's functions T^0 and all retarded, advanced and lesser Green's functions $N_{a\pm\sigma}$ should be replaced by the corresponding pseudo-particle Green's functions $N_{\pm\sigma}$ with corresponding changing of energy variables $E_{T^0} \to 2\varepsilon + U$, $\varepsilon_a \to \varepsilon$. For calculation of tunneling current and zero frequency noise in $T^0 - a$ channel we determine pseudo particle Green's functions $T^{0<} = -N^{T^0}(T^{0A} - T^{0R})$, $N^{<}_{a\pm\sigma} = N^a(N^{0A}_{a\pm\sigma} - N^{0R}_{a\pm\sigma})$ and $T^{0R(A)} = \frac{1}{\omega - E_{T^0} \pm i\gamma^{T^0 a}}$, $N^{0R(A)}_{a\pm\sigma} = \frac{1}{\omega - \varepsilon_a \pm i\gamma^{T^0 a}}$. The pseudo particle occupation numbers can be found from kinetic equations shown in **Appendix I**:

$$
\begin{aligned}
N^{T^0} &= \frac{N_T^{T^0 a}}{2 - N_T^{T^0 a}}, \\
N^a &= \frac{1 - N_T^{T^0 a}}{2 - N_T^{T^0 a}},
\end{aligned}
\tag{31}
$$

where $N_T^{T^0 a} = \frac{\gamma_L^{T^0 a}\Phi_k^{T^0 a} + \gamma_R^{T^0 a}\Phi_p^{T^0 a}}{\gamma_L^{T^0 a} + \gamma_R^{T^0 a}}$ and $N_T^{T^0 a} \equiv N_T(E_{T^0} - \varepsilon_a)$. The expression for the tunneling current reads

$$
I_\sigma^{T^0 a} = \frac{4\gamma_L^{T^0 a}\gamma_R^{T^0 a}\left(\Phi_k^{T^0 a} - \Phi_p^{T^0 a}\right)}{\gamma^{T^0 a}\left(2 - N_T^{T^0 a}\right)},
\tag{32}
$$

where $\gamma^{T^0 a} = \gamma_L^{T^0 a} + \gamma_R^{T^0 a}$.

Similarly one can obtain the expression for the zero frequency noise spectra. The leading terms can be obtained from diagrams shown in Fig.3 of the main text of the paper. Quantum corrections contain maximally crossing diagrams, which are depicted in Fig.4 of the main text of the paper. Fixing the sign of spin index σ for the leads Green function $J^{<}_{k\sigma}(\omega)$ automatically determines the signs of spin index σ for all pseudo-particle Green's functions $N_{\pm\sigma}$ and electron Green's functions in the leads $J_\sigma^{T<(>)}$ in maximally crossing diagrams. The expression for the total zero frequency noise

$$
S^{T^0 a}(0) = \sum_\sigma S^\sigma_{T^0 a}(0) = 2\frac{4\gamma_L^{T^0 a}\gamma_R^{T^0 a}}{\gamma^{T^0 a}(2 - N_T^{T^0 a})}\left(1 - \frac{2\gamma_L^{T^0 a}\gamma_R^{T^0 a}}{(\gamma^{T^0 a})^2}\right)\left[(\Phi_k^{T^0 a})^2(1 - \Phi_p^{T^0 a})^2 + (k \leftrightarrow p)\right].
\tag{33}
$$

The expressions for tunneling current and zero frequency noise for $S^0 - S - 0$ channel can be obtained analogously to the case of tunneling for a single dot with $U \neq 0$ taking into account both contributions from diagrams with $D - N_{\pm\sigma}$ and $N_{\pm\sigma} - B$ pseudo-particle Green's functions. The following changes should be done: $S^0 \to D$, $N_{s\pm\sigma} \to N_{\pm\sigma}$ and corresponding changing of energy variables $E_{S^0} \to 2\varepsilon + U$, $\varepsilon_S \to \varepsilon$. The pseudo-particle Green's function corresponding to the empty state remains unchanged.

For calculation of tunneling current and zero frequency noise in $S^0 - S - 0$ channel we determine pseudo-particle Green's functions $S^{0<} = -N^{S^0}(S^{0A} - S^{0R})$, $N^{<}_{S\pm\sigma} = N^S(N^{0A}_{S\pm\sigma} - N^{0R}_{S\pm\sigma})$ and $S^{0R(A)} = \frac{1}{\omega - E_{S^0} \pm i\gamma^{S^0 S}}$, $N^{0R(A)}_{S\pm\sigma} = \frac{1}{\omega - \varepsilon_a \pm i\gamma^{S^0 S}}$. One should also introduce functions $B^{R(A)} = \frac{1}{\omega \pm i\gamma}$, $B^< = b(B^R - B^A)$. The pseudo-particle occupation numbers can be found from kinetic

equations shown in **Appendix I**.

$$N^{S^0} = \frac{N_T^S N_T^{S^0 S}}{1+N_T^S - N_T^{S^0 S}},$$
$$N^S = \frac{N_T^S(1-N_T^{S^0 S})}{1+N_T^S - N_T^{S^0 S}},$$
$$b = \frac{(1-N_T^S)(1-N_T^{S^0 S})}{1+N_T^S - N_T^{S^0 S}}, \quad (34)$$

where $N_T^{S^0 S} = \frac{\gamma_L^{S^0 S}\Phi_k^{S^0 S}+\gamma_R^{S^0 S}\Phi_p^{S^0 S}}{\gamma_L^{S^0 S}+\gamma_R^{S^0 S}}$, $N_T^S = \frac{\gamma_L^S\Phi_k^S+\gamma_R^S\Phi_p^S}{\gamma_L^S+\gamma_R^S}$ and $N_T^{S^0 S} \equiv N_T(E_{S^0}-\varepsilon_S)$, $N_T^S \equiv N_T(\varepsilon_S)$. Functions $\Phi_{k(p)}^{S^0 S} \equiv \Phi_{k(p)}(E_{S^0}-\varepsilon_S)$ and $\Phi_{k(p)}^S \equiv \Phi_{k(p)}(\varepsilon_S)$ are determined by Eq.(17). The corresponding pseudo-particle occupation numbers d, n and b could be represented by N^{S^0}, N^S and b determined from kinetic equations shown in **Appendix I**.

Tunneling current in $S^0-S^{\pm}-0$ channels can be calculated using diagrams shown in Fig.5 of the main text of the paper. For each spin projection tunneling current reads

$$I_\sigma^{S^0-S-0} = I_\sigma^{S^0-S} + I_\sigma^{S-0} = \frac{4\gamma_L^{S^0 S}\gamma_R^{S^0 S}}{\gamma^{S^0 S}(1+N_T^S-N_T^{S^0 S})}N_T^S(\Phi_k^{S^0 S}-\Phi_p^{S^0 S}) + \frac{4\gamma_L^S\gamma_R^S}{\gamma^S(1+N_T^S-N_T^{S^0 S})}(1-N_T^{S^0 S})(\Phi_k^S-\Phi_p^S), \quad (35)$$

where $\gamma^S = \gamma_L^S+\gamma_R^S$ and $\gamma^{S^0 S} = \gamma_L^{S^0 S}+\gamma_R^{S^0 S}$. Calculation details are shown in **Appendix II**. Similarly, the zero frequency noise also has two contributions from S^0-S and $S-0$ channels. The leading terms in zero frequency noise for both contributions are shown in diagrams depicted in Fig.6 of the main text of the paper. The quantum corrections contain the maximally crossing diagrams shown in Fig.7 of the main text of the paper. Fixing the sign of spin index σ for the leads Green's function $J_{k\sigma}^{<}(\omega)$ automatically determines the signs of spin index σ for all pseudo particle Green's functions $N_{\pm\sigma}$ and electron Green's functions in the leads $J_\sigma^{T<(>)}$ in maximally crossing diagrams.

$$S_\sigma^{S^0-S-0}(0) = I_\sigma^{S^0-S}\left(1-\frac{2\gamma_L^{S^0 S}\gamma_R^{S^0 S}}{(\gamma^{S^0 S})^2}\right) + I_\sigma^{S-0}\left(1-\frac{2\gamma_L^S\gamma_R^S}{(\gamma^S)^2}\right). \quad (36)$$

References

1. Maslova, N. S., Arseyev, P. I. & Mantsevich, V. N. Collective spin correlations and entangled state dynamics in coupled quantum dots. *Phys. Rev. E* **97**, 022135, DOI: 10.1103/PhysRevE.97.022135 (2018).
2. Arseyev, P. I., Maslova, N. S. & Mantsevich, V. N. Charge and spin configurations in the coupled quantum dots with coulomb correlations induced by tunneling current. *The Eur. Phys. J. B* **85**, 410, DOI: 10.1140/epjb/e2012-30579-x (2012).
